# Profiles of global mutations in the human intercellular adhesion molecule-1 (ICAM-1) shed light on population-specific malaria susceptibility

**DOI:** 10.1186/s12864-023-09846-9

**Published:** 2023-12-13

**Authors:** Jasmita Gill, Himmat Singh, Amit Sharma

**Affiliations:** 1 ICMR-National Institute of Malaria Research, Sector-8 Dwarka, New Delhi, India; 2 International Centre for Genetic Engineering and Biotechnology, New Delhi, India

**Keywords:** Malaria, PfEMP1, ICAM-1, SNP, Structural analysis

## Abstract

*Plasmodium falciparum* is responsible for malaria-related morbidity and mortality. PfEMP1 (*P. falciparum* erythrocyte membrane protein 1) mediates infected erythrocytes adhesion to various surface vascular receptors, including intercellular adhesion molecule-1 (ICAM-1), associating this interaction with severe malaria in several studies. Genetic variation in host ICAM-1 plays a significant role in determining susceptibility to malaria infection via clinical phenotypes such as the ICAM-1^Kilifi^ variant which has been reported to be associated with susceptibility in populations. Our genomic and structural analysis of single nucleotide polymorphisms (SNPs) in ICAM-1 revealed 9 unique mutations each in its distinct A-type and BC-type PfEMP1 DBLβ-interacting regions. These mutations are noted in only a few field isolates and mainly in the African/African American population. The ICAM-1^Kilifi^ variant lies in a flexible loop proximal to the DBLβ-interacting region. This analysis will assist in establishing functional correlations of reported global mutations via experimental and clinical studies and in the tailored design of population-specific genetic surveillance studies. Understanding host polymorphism as an evolutionary force in diverse populations can help to predict predisposition to disease severity and will contribute towards laying the framework for designing population-specific personalized medicines for severe malaria.

**Supplementary Information:**

The online version contains supplementary material available at 10.1186/s12864-023-09846-9.

## Background

*Plasmodium falciparum* is primarily responsible for malaria-related morbidity and mortality worldwide as it causes life-threatening forms of the disease [1]. Upon malaria infection, *P. falciparum* antigens on the surface of infected erythrocytes (IEs) in the human host mediate cytoadherence by binding to various host receptors in different tissues and organs of infected individuals to avoid elimination by the spleen. *P. falciparum* erythrocyte membrane protein 1 (PfEMP1) family, coded by ~ 60 members of highly variable *var* genes, are classified as A, B, and C, consisting of different combinations of 3–10 Duffy-binding like (DBL) domains and cysteine-rich interdomain regions (CIDR) that form conserved domain cassettes within the *P. falciparum* genome [2]. The extracellular domain of PfEMP1 mediates the adhesion with specific endothelial receptors ICAM-1, EPCR, CD36, and CSA and plays a prominent role in the pathogenesis of severe and cerebral malaria as this sequestration leads to circulatory disturbances and inflammation affecting one or more organs leading to severe disease and fatal complications [3–5].

Intercellular adhesion molecule-1 (ICAM-1), a member of the immunoglobulin superfamily is a heavily glycosylated transmembrane protein and is present in membranes of leukocytes and endothelial cells [6]. It is known to play a role in inflammatory processes and in T-cell-mediated host defence system as it facilitates various immune responses requiring intercellular contact and collaboration. Binding to ICAM-1 has been established to be crucial and suspected to play an important role in cerebral malaria as it is involved in the selective accumulation of infected erythrocytes in the brains of cerebral malaria patients [2, 6–13]. The subclasses of DBLβ domains found in A, B, and C PfEMP1 are known to bind ICAM-1 [2, 3]. Studies show that parasites which express PfEMP1 that binds to both ICAM-1 and endothelial protein C receptor (EPCR) (via the neighbouring CIDR domain), are possibly associated with an increased risk of developing cerebral symptoms of *P. falciparum* infections [14–16].

Genetic polymorphism in both erythrocyte surface antigen PfEMP1 and host receptor ICAM-1, possibly inherited or due to eco-environmental factor(s), can affect disease outcomes causing severity in many patients. Thus, the impact of host and parasite genetic variations is pivotal to host-parasite interaction in context with predisposition to severe disease and has implications for the design of treatments and development of PfEMP1-based vaccines [3, 4, 15]. Host genetic polymorphisms can be responsible in modulating vaccine-induced immune response on different scales and this information assists in generating vaccine strategies to optimize the antibody response and help understand the development of immunity towards severe malaria in individuals after repeated infections. Polymorphism studies in host ICAM-1 via gene sequencing conducted in field isolates collected from malaria patients have shown conflicting clinical manifestations. Studies from Ghana and Uganda show the association of ICAM-1 with cerebral malaria [11–13], however, no association was reported from Benin and Thailand [17–19]. ICAM-1^Kilifi^ variant (K56M; updated dbSNP ID: rs5491) identified in the Kilifi region of Kenya was found to be more frequent in patients suffering from cerebral malaria compared to controls, thereby clinically establishing high susceptibility to cerebral malaria [20]. Functional analysis of ICAM-1^Kilifi^ revealed the distinctive nature of the interaction between infected erythrocytes and ICAM-1 in different Pf field isolates and reduced binding in the ICAM-1^Kilifi^ variants [21]. However, in other field isolates from Gabon an association was not seen and instead, it led to protection from severity while an association could not be established in studies from Benin, Gambia, Kenya, Malawi, Indonesia, Tanzania, and Thailand [22–26].

In this work, we have collated 347 single nucleotide polymorphisms (SNPs) in ICAM-1 from dbSNP that covers independent studies and more than 76,156 field isolates. We structurally mapped the SNPs onto the three-dimensional structures of two complexes of ICAM-1 and A-type and BC-type PfEMP1 DBLβ domains. Both structures exhibit an overall similar binding architecture, however, ICAM-1-binding residues of A-type and BC-type are distinct and our analysis revealed 9 unique mutations in the DBLβ-interacting residues of ICAM-1 in each structure. These mutations are seen in only 1 or 2 field isolates and mainly in one or two populations. The clinically-established ICAM-1^Kilifi^ variant known to be associated with disease susceptibility lies in a flexible loop proximal to the DBLβ-interacting region. Our work provides systematic genomic and structural insights into ICAM-1 global mutations in context with the likelihood of altered malaria susceptibility across populations. This work will assist in assessing the functional association of known mutations via experimental and clinical validation studies, and plan population-wide genetic surveillance via epidemiological studies to make correlations with disease susceptibility and likely predisposition in specific populations. This understanding will help in designing personalized population-specific treatments and may help tackle the burden of disease severity due to malaria.

## Methods

### SNP data

Non-synonymous mutations (missense) and the corresponding 347 amino acid substitutions in ICAM-1 (1-542 aa sequence length; Gene ID: “ICAM1”) were collated from the public domain archive - Single Nucleotide Polymorphism Database (dbSNP) [27] (Supplementary Table 1). Population-wide data was collated from The Genome Aggregation Database (gnomAD) v3.1 release consisting of 76,156 whole genomes using GRCh38 reference (www.gnomad.broadinstitute.org) [28] by using Gene ID “ICAM1”. Population classification is available at www.gnomad.broadinstitute.org/help/what-populations-are-represented-in-the-gnomad-data. For ease of visualization, pie charts for population analysis were prepared using the number of field samples in which mutations were seen instead of absolute frequencies. The occurrence frequency of an SNP (in percentage) is calculated as:

$$\frac{{number{\text{ }}of{\text{ }}field{\text{ }}samples{\text{ }}that{\text{ }}contain{\text{ }}alternate{\text{ }}alleles}}{{total{\text{ }}number{\text{ }}of{\text{ }}field{\text{ }}samples}} \times 100$$

The SNP frequency per domain of ICAM-1 (in percentage) is calculated as:

$$\frac{{number{\text{ }}of{\text{ }}SNPs{\text{ }}in{\text{ }}a{\text{ }}domain}}{{total{\text{ }}number{\text{ }}of{\text{ }}residues{\text{ }}in{\text{ }}that{\text{ }}domain}} \times 100$$

### Sequence and structural analysis

Three-dimensional crystal structures of ICAM-1 and PfEMP1 DBLβ domain complex were accessed from Protein Data Bank (PDB; www.rcsb.org; PDB IDs: 5MZA and 6S8U; *P. falciparum* strains 3D7 and IT4var13, respectively) [29, 30]. The ICAM-1 sequence in the PDB structures is offset by -28 in comparison with the UniProt entry (https://www.uniprot.org/uniprotkb/P05362/entry) when using Gene ID “ICAM1” and thus UniProt/dbSNP sequence numbering of ICAM-1 was taken as final. The interacting partners of ICAM-1 and PfEMP1 DBLβ domain are collated from PDBsum (http://www.ebi.ac.uk/pdbsum/) and structural analysis was depicted using Pymol (www.pymol.org).

## Results and discussion

### SNP profile and structural mapping of ICAM-1 SNPs

**Fig. 1:**
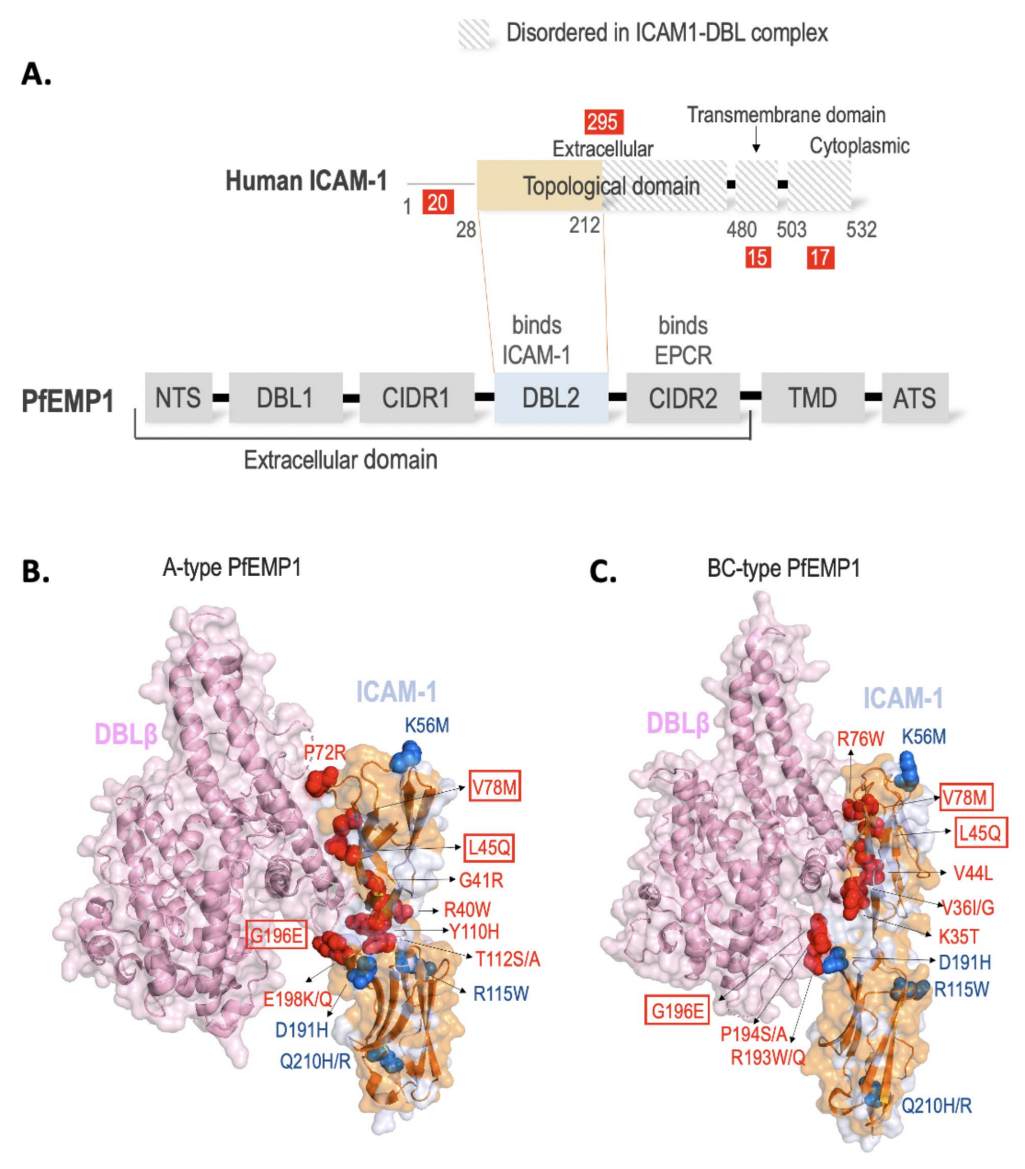
Human intercellular adhesion molecule-1 (ICAM-1) and *Plasmodium falciparum* erythrocyte membrane protein 1 (PfEMP1). **(A)** Domain diagram of ICAM-1 and PfEMP1. Human ICAM-1: Topological domain is colored brown and disordered transmembrane domain is shown in pattern. PfEMP1: DBL2 domain is colored blue. The total number of mutations in different domains of ICAM-1 are shown in red box **(B)** A-type PfEMP1 DBLβ in complex with ICAM-1 (PDB ID: 5MZA) **(C)** BC-type PfEMP1 DBLβ in complex with ICAM-1 (PDB ID: 6S8U). ICAM-1 and DBLβ are colored light blue and pink respectively and shown as ribbons and transparent surface. All 116 SNPs in ICAM-1’s structurally ordered topological domain are colored orange. SNPs that lie in the DBLβ-interacting region are shown as red spheres. The high-frequency SNPs are shown as blue spheres. The common interacting residues between A-type and BC-type are encircled in a red box

Our analysis revealed a total of 347 unique non-synonymous single nucleotide polymorphisms (SNPs) in human host receptor ICAM-1, of which; 20 are in the N-terminal (71% SNP frequency per domain), 295 in the extracellular topological domain (65% frequency), 15 in the transmembrane domain (62.5% frequency) and 17 in the cytoplasmic topological domain (57% frequency) (Fig. 1A, Supplementary Table 1). Of the 347 mutations, 116 mutations that lie in the topological domain could be mapped onto the known three-dimensional complex structures of ICAM-1 and PfEMP1 DBLβ domain as the remaining residues in the available structures are disordered (Fig. 1A). ICAM-1 consists of five immunoglobin (Ig)-like domains D1 to D5 and the two ICAM-1 complexes with DBLβ domain contain ICAM-1^D1D2^ referred to as ICAM-1 from hereon [27, 28]. Structural mapping of 116 ICAM-1 mutations in the topological domain revealed a uniform spread of mutations with multiple clusters (Fig. 1B, C).

### SNPs and their effect on the interaction profiles of ICAM-1 with PfEMP1 DBLβ domain

#### A-type PfEMP1

**Fig. 2:**
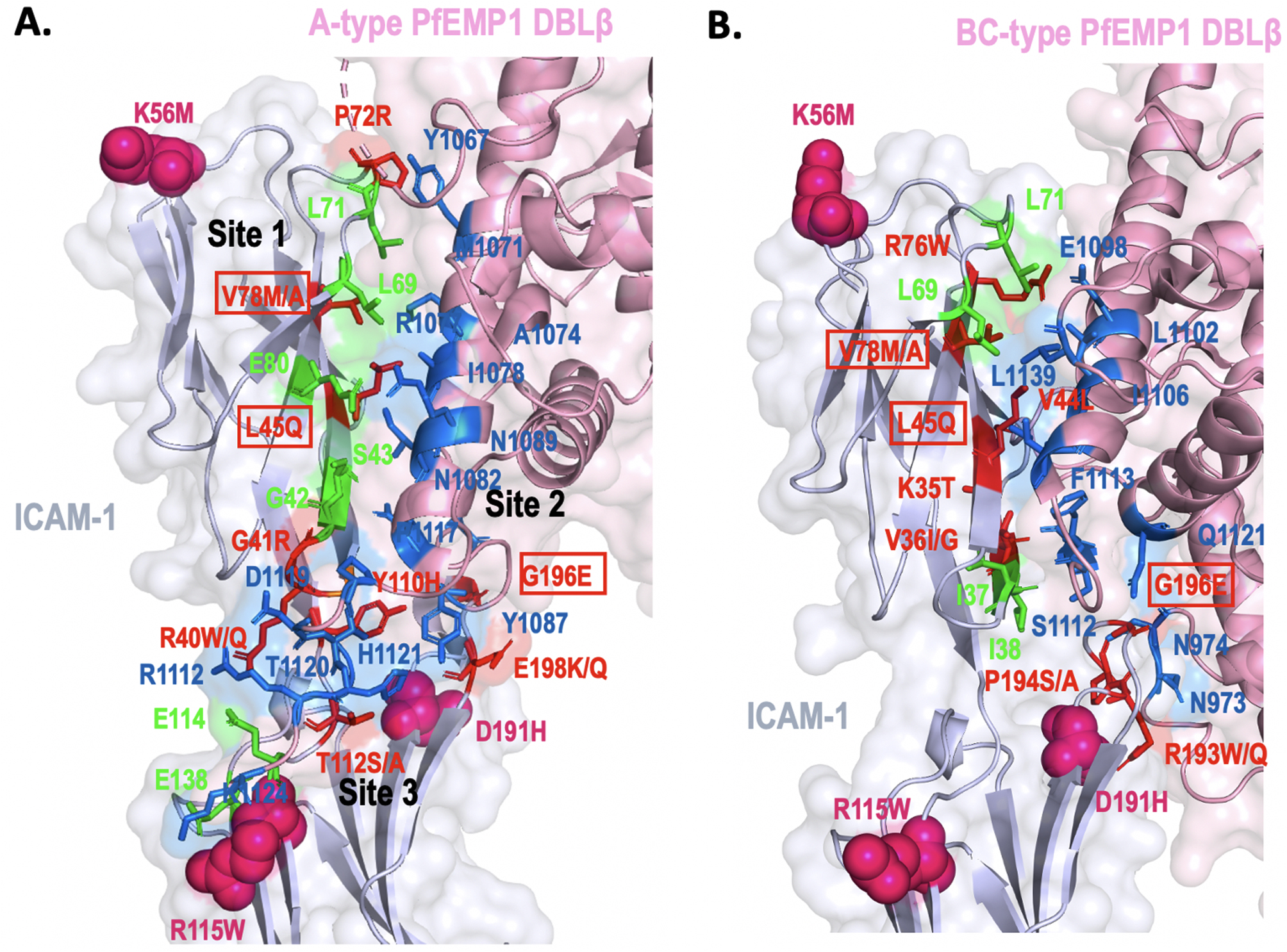
Mapping of ICAM-1 SNPs on the three-dimensional complexes of PfEMP1 DBLβ and ICAM-1 **(A)** A-type PfEMP1 **(B)** BC-type PfEMP1. ICAM-1 is colored grey blue and DBLβ is colored pink and shown as ribbon and transparent surface. Interacting residues of ICAM-1 are shown as red (SNP present) or green (no SNP) sticks and ICAM-1-interacting DBLβ residues are shown as blue sticks for A-type and BC-type (*P. falciparum* 3D7 and IT4var13 strains (PDB ID: 5MZA and 6S8U)). The high-frequency ICAM-1 mutations that lie away from the binding interface are shown as pink spheres. The common ICAM-1-binding residues between A-type and BC-type are encircled in a red box. ICAM-1 interacting subsites 1, 2 and 3 are marked for A-type PfEMP1.

**Fig. 3:**
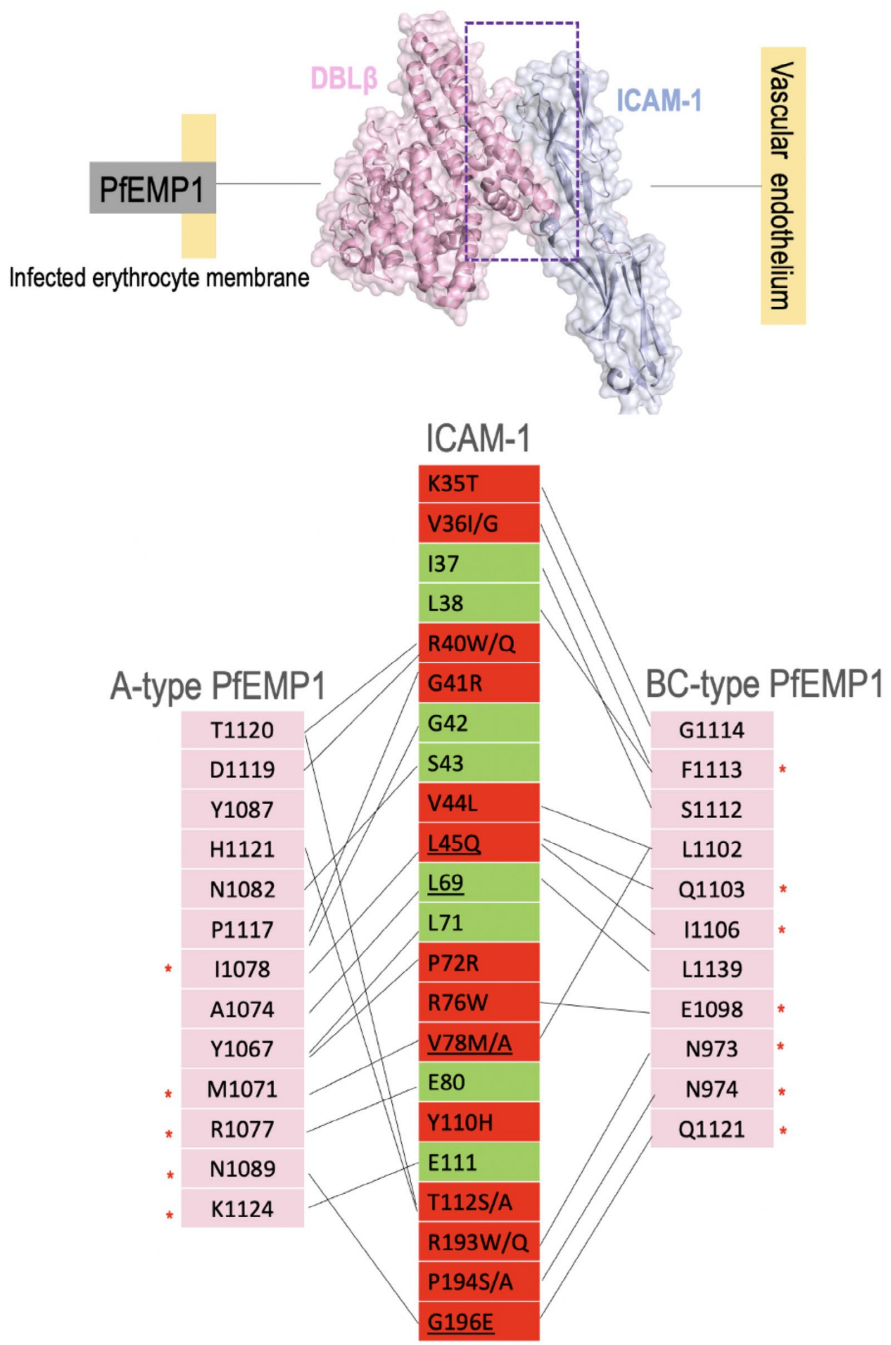
Interactome of ICAM-1 and A-type/BC-type PfEMP1 DBLβ. ICAM-1 (Ig-like domains D1 and D2) and DBLβ are colored grey blue and pink respectively and shown as ribbons with transparent surface. ICAM-1 binding residues of A-type and BC-type are shown in pink boxes. The residues which show variation based on 145 predicted ICAM-1-binding DBLβ sequences [29] are marked with a red asterisk. DBLβ-interacting residues of ICAM-1 are shown in green or red boxes, no mutation and having mutation, respectively. The common ICAM-1 residues involved in interaction with both A-type and BC-type are underlined

A total of 9 mutations are present in the A-type PfEMP1 DBLβ-interacting region of ICAM-1 (Figs. 1A and 2A). In an earlier analysis, three subsites with critical ICAM-1 interacting residues were identified in the A-type PfEMP1 DBLβ as their disturbance could disrupt the interaction [29] (Figs. 2A and 3). The first subsite constitutes A-type DBLβ residues M1071, R1077, and I1078 that interact with ICAM-1 residues L45 (involved in hydrophobic interaction, mutation L45Q), V78 (hydrophobic interaction, conservative mutation V78M/A, 1 European (non-Finnish) sample), and E80 (involved in h-bond, no mutation). Analysis indicates no likely effect of these ICAM-1 mutations on the interaction with DBLβ domain (Fig. 2A). Interestingly, R1077A and I1078A mutations have been experimentally shown to disrupt binding with ICAM-1 [29]. Also, based upon 145 A-type DBLβ and 10 BC-type DBLβ sequences earlier known/predicted that could bind to ICAM-1 (using DBLβ sequence motif (I[V/L]x_3_N[E]GG[P/A]xYx_27_GPPx_3_H), I1078 is a highly conserved position [29]. The second subsite constitutes A-type DBLβ residues N1082 and N1089 which interact with ICAM-1 S43 (involved in h-bond, no mutation) and G196 (h-bond, mutation G196E, 13 African/African American samples), respectively. At this subsite, N1082 is thought to be conserved, intriguingly, mutation N1082A has been experimentally shown to disrupt ICAM-1 binding [29]. G196E mutation could have a likely effect as it may modify the interaction by possibly increasing the binding affinity. G196E mutation has been reported mainly from the African/African American population. The third subsite constituting A-type DBLβ residues T1120, H1121, and K1124 interacts with ICAM-1 residue R40 (involved in h-bond, mutation R40W/Q, 16 Latino/Admixed American samples and 1 European), T112 (hydrophobic interaction, mutation T112S/A, 1 Latino/Admixed American sample), and E114 (hydrophobic, no mutation), respectively (Figs. 2A and 3). This subsite forms part of an intrinsically disordered loop (aa 1115–1124) which becomes partially ordered in the A-type DBLβ when bound to ICAM-1 versus the unbound form [29]. ICAM-1 mutation R40W/Q at this subsite could alter the affinity of binding since it is involved in h-bonding with DBLβ T1120 (Figs. 2A and 3). Interestingly, all three DBLβ residues, T1120, H1121, and K1124 are seen to be conserved based on 145 predicted ICAM-1-binding DBLβ sequences, however, H1121A mutation has been experimentally shown to disrupt ICAM-1 binding [29]. Additional sequencing of field isolates of parasite DBL domains is needed to interpret sequence conservation or variation in the ICAM-binding region of the A-type DBLβ domain.

In addition, since the ICAM-1-binding region of A-type DBLβ is described as conserved [29], other ICAM-1 mutations like G41R, P72R, Y110H, and E198K/Q can significantly alter the interaction of ICAM-1 with PfEMP1 by disrupting h-bonds and salt bridges (mutation Y110H) as well as by changing the electrostatic/hydrophobic surfaces (G41R, P72R, Y110H, and E198K/Q) of the binding site (Figs. 2A and 3). G41R is seen in only 1 European sample and E198K/Q is seen in 1 African/African American sample, while for other mutations population-wide data is unavailable. It is clear that the interacting region mutations of ICAM-1 are present in very few field isolates. ICAM-1^Kilifi^ mutation (K56M) lies in a flexible loop proximal to the interaction region (Fig. 1). Thus, despite predicted sequence conservation in A-type DBLβ, mutations in its host interacting partner ICAM-1 have implications for binding affinity and altered disease susceptibility in patients infected with parasites having the A-type PfEMP1. A neighbouring domain of A-type DBLβ, the CIDRα1 binds to a different receptor, endothelial protein C receptor (EPCR) and this association is implicated in severe childhood malaria [15]. CIDRα1 is highly polymorphic as has been seen in field isolate studies. The complex interplay between parasites and EPCR based on global mutations has been described earlier [16].

#### BC-type PfEMP1

A related three-dimensional structure of the BC-type PfEMP1 bound to ICAM-1 revealed an overall binding orientation and architecture similar to the A-type, however, the binding site residues of ICAM-1 differ in both as only 4 DBLβ-interacting residues of ICAM-1 out of total 16 are common between the A-type and BC-type [29, 30]. Overall, 9 mutations are seen in the interacting region with DBLβ of which only three mutations V78M/A, L45Q, and G196E are common with the A-type (Figs. 2B and 3). Based on 145 predicted ICAM-1-binding DBLβ sequences, ICAM-1 interacting residues of BC-type DBLβ are shown to be not conserved (DBLβ N973, N974, E1098, Q1103, F1113, Q1121, L1139) in comparison to the A-type [29, 30].

Three critical regions have been described for BC-type DBLβ and ICAM-1 interaction [30]. The first region is dominated by h-binding interactions (Figs. 2B and 3). DBLβ mutation Q1103A causes a 200-fold decrease in binding affinity with ICAM-1, and it is involved in h-bonding with ICAM-1 residue L45 (mutation L45Q) (Figs. 2B and 3) [30]. L45 is one of the three overlapping DBLβ-interacting residues in A-type and BC-type (Fig. 2). Secondly, a significant hydrophobic patch on DBLβ, which constitutes hydrophobic residues L1102, I1106, and F1113, plays an important role in the binding as mutations in these residues led to a 200-fold reduced affinity [30]. I1106 makes hydrophobic interactions with ICAM-1 L45 (mutation L45Q), and L1102 and F1113 are involved in hydrophobic interactions with ICAM-1 residues V36I/G (mutation V36I/G), L38, V44 (conservative mutation V44L), L71, and V78 (mutation V78M/A). It is noted that V78 is one of 3 residues that are common between A-type and BC-type’s ICAM-1 interaction. Additionally, a β-sheet augmentation interaction is specifically seen in BC-type where S1112 and G1114 interact with ICAM-1 K35 and I37, in which mutation is seen only in one residue (K35T). Further, 3 glycines in DBLβ, G1114 (involved in h-bond with ICAM-1 K35), G1115, and G1116 were mutated in an earlier study but no effect was seen on interaction with ICAM-1 [30]. Lastly, similar to the A-type, an intrinsically disordered loop (aa 1107–1117) in BC-type becomes partially ordered when binding with ICAM-1 occurs. P1117 at one end of this loop is involved in hydrophobic interaction with ICAM-1 (Figs. 2B and 3).

### Population-wide distribution of ICAM-1 mutations

**Fig. 4:**
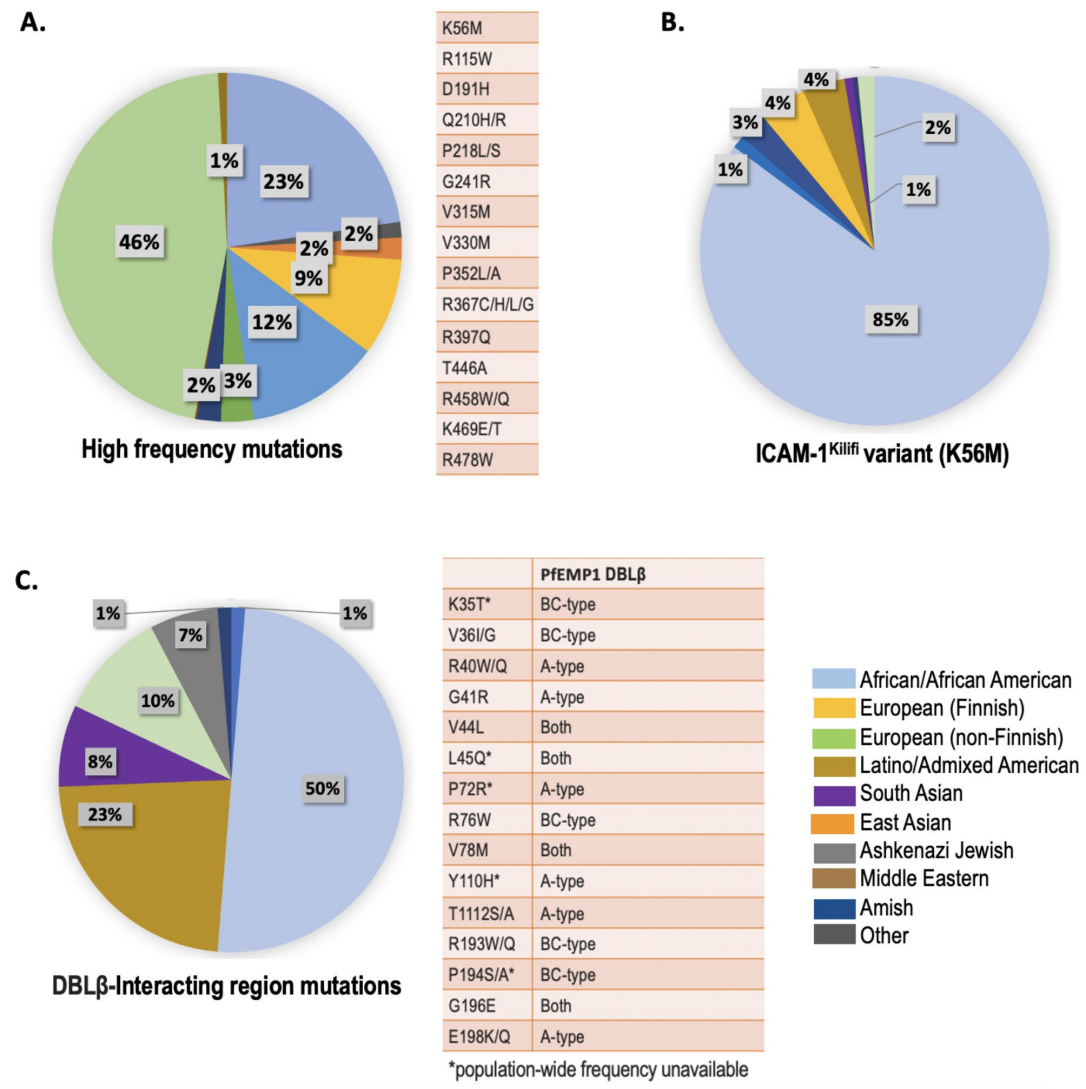
Population-wide distribution of SNPs in human host ICAM-1. The pie-charts show population-wide percentage distribution of mutations where data is available from gnomAD (www.gnomad.broadinstitute.org). **(A)** High-frequency mutations (defined as mutations seen in more than 100 field isolates) **(B)** ICAM-1^Kilifi^ (K56M) variant **(C)** A-type and BC-type DBLβ-interacting region mutations of ICAM-1

The population-wide distribution of all 347 mutations in ICAM-1 from gnomAD (where available) shows that mutations in this human receptor occur more commonly in African/African American, European (non-Finnish), and Latino/Admixed American populations. 15 high-frequency mutations (defined as occurring in > 100 field isolates) are present majorly in the European (non-Finnish) population followed by African/African American, Latino/Admixed American along with low occurrences in other populations (Fig. 4A). Four of these 15 high-frequency mutations could be mapped onto the structures while the remaining 11 are disordered, thus, no structural inference could be made (Figs. 1 and 2). Only 1 mutation Q210H, lies within 5Å of the ICAM-1 DBLβ-interacting region as the other 3 lie in distant regions. ICAM-1^Kilifi^ mutation, clinically established to impart high-risk susceptibility to cerebral malaria or alternately offer protection, occurs in high frequency. ICAM-1^Kilifi^ is seen mainly in the African/African American population (85% samples) followed by European (non-Finnish) (25% samples) and in low occurrences ( < = 4%) in other populations (Fig. 4B). Intriguing, the mutations that lie in the A-type and BC-type DBLβ-interacting regions of ICAM-1 are seen in low frequencies or the population data is unavailable from gnomAD. The mutations where population-wide data is available (V46I, R40W/Q, V44L, R76W, V78M/A, T112S/A, R193W, and G196E), occur in 1 or 2 samples within a particular population and 50% of these mutations are seen in the African/African American population followed by Latino/Admixed American, and low frequencies in European (non-Finnish), South Asian and Amish populations (Fig. 4C). Three ICAM-1 mutations in the DBLβ-interacting regions which are common to both A-type and BC-type occur in a low number of samples (V78M/A, 1 European (non-Finnish) sample); (G196E, 13 African/African American samples) and L45Q (data unavailable).

### Implications of ICAM-1 global mutations on vaccine design and genetic surveillance

The two three-dimensional structures of ICAM-1 in complex with the DBLβ domain of PfEMP1 show that despite significant sequence differences, the position of key residues and the length of key sequences in the ICAM-1-binding region of the evolutionary distinct A-type and BC-type are similar since the core binding region in both structures consists of a hydrophobic patch, along with h-bonding and similar partially ordered loops in their ICAM-1 bound form [29, 30]. These myriad interactions are responsible for enhancing structural stability that leads to tighter adherence and non-clearance by the spleen. The A-type PfEMP1 is described as significantly less diverse making it a strong vaccine candidate against cerebral malaria [29]. However, the same study states that such a conserved host receptor-binding site is an exception as it is more advantageous for the parasite to have sequence variation in its human host partner interaction footprint, but at the same time retain the overall binding architecture in other variants, for example the more diverse BC-type. This scenario can efficiently retain the binding capability (albeit with a different set of residues) and withstand immune pressure including that of a vaccine. On these lines, CIDR, the neighbouring domain of DBL on the PfEMP1, which binds to human endothelial protein C receptor (EPCR), exhibits high sequence variability (based on 885 field isolates) but has an overall conserved structural architecture in its EPCR-binding region [16, 31]. ICAM-1 and EPCR are of significant interest as several studies have strengthened their potential dual role in the pathogenesis of cerebral malaria [14, 15].

PfEMP1 is highly polymorphic from patient to patient even among one particular population at one endemic site from where field isolates have been collected. This suggests that PfEMP1-based vaccines will be challenging and susceptible to population variants. Our analysis reiterates that co-existence of high sequence variation in the parasite PfEMP1 and human host ICAM-1 requires population-wide genetic surveillance studies to understand the implications for altered susceptibility and/or disease severity. We show high sequence variability in the A-type and BC-type PfEMP1 DBLβ-interacting region of ICAM-1 based on reported global mutations from independent studies. Earlier studies have analysed PfEMP1 DBLβ sequences known/predicted to bind ICAM-1 and experimental data shows A-type DBLβ mutations I1078S, R1077A, H1121A, N1082A, P1116 and G1083A and BC-type DBLβ Q1103A, L1102, I1106, and F1113 to alter binding with ICAM-1 [29, 30]. Thus, mutations in both host ICAM-1 and parasite PfEMP1 DBLβ are likely to play a role in altering the interaction. These understandings will propel functional assessment of reported mutations to decipher their biological significance and will assist in designing personalized treatments for malaria patients. Understanding the direct and indirect structural impact of host mutations (in the interacting region and elsewhere) which can likely alter susceptibility to malaria by changing the ICAM-1 and DBLβ interaction is critical in the context of deciphering disease susceptibility.

For example, the hypothesis of ICAM-1^Kilifi^ variant needs to be further explored in populations to understand if this mutation increases or decreases the severe disease risk possibly due to higher cytoadherence or other factors. An understandable possibility is the decreased ability of ICAM-1-binding parasites to adhere to the endothelium via ICAM-1 which would cause a reduction in parasite multiplication and reduced disease severity [21]. However, an opposite effect would lead to high predisposition to severe malaria [21]. Interestingly, the loop consisting this mutation appears to be involved in several interactions [21] (Fig. 1). The involvement of ICAM-1 in two important features of the immune response has been explored, (i) loss of fibrinogen binding by ICAM-1^Kilifi^ variants (ligand of ICAM-1 for different physiological functions), and (ii) reduced LFA-1 mediated adhesion of leukocytes in the ICAM-1^Kilifi^ variant resulting in a beneficial immunomodulatory effect [21]. Understandably, cohort studies on cerebral malaria are required with a focus on genetic surveillance of host factor mutations along with parasite genes that drive cytoadhesion [16]. Comprehensive data on global mutations can assist in designing experimental and clinical studies to validate the biological significance of mutations in relation to disease severity by possibly linking specific mutations in ICAM-1 with altered malaria susceptibility in particular host populations.

## Conclusions

During malaria infection by *Plasmodium falciparum*, PfEMP1 DBL (Duffy-binding like) domains on infected erythrocytes (IEs) can adhere to surface vascular receptor intercellular adhesion molecule-1 (ICAM-1), and this association is implicated in the pathogenesis of severe malaria including cerebral malaria. Host genetic variation in ICAM-1 can lead to altered disease susceptibility and clinical outcome, including variant ICAM-1^Kilifi^ which is associated with higher or sometimes in contradiction, decreased susceptibility to cerebral malaria in different geographical regions. In this work, we present detailed genomic and structural analyses of ICAM-1 single nucleotide polymorphisms (SNPs) from dbSNP. Structural mapping revealed 9 distinct ICAM-1 mutations in both A-type and BC-type PfEMP1 DBLβ-binding residues that occur in low frequency in only 1 or 2 field isolate samples. The high-frequency ICAM-1^Kilifi^ mutation lies in a flexible loop proximal to the key binding region. This analysis will assist in facilitating functional correlations of reported mutations via targeted experimental and clinical validation studies. The data will also help in designing population-specific genomic surveillance studies. Understanding host polymorphism in populations as an evolutionary force can help to predict susceptibility and predisposition to severe disease. Epidemiological studies that track the outcomes of malaria infections and then assess the genomic markers in the host and the parasite will be critical in laying a framework for personalized medicine for severe malaria.

### Electronic supplementary material

Below is the link to the electronic supplementary material.

Supplementary Material 1

Supplementary Material 2

## Data Availability

The dataset(s) supporting the conclusions of this article are publicly available at the Single Nucleotide Polymorphism Database (dbSNP) (www.ncbi.nlm.nih.gov/snp/) and The Genome Aggregation Database (gnomAD) (www.gnomad.broadinstitute.org). The analysis material as Microsoft Excel files from this study are available from the corresponding author upon request.
