# Supplementary material for: Profiles of global mutations in the human intercellular adhesion molecule-1 (ICAM-1) shed light on population-specific malaria susceptibility
Source: BMC Genomics. 2023 Dec 13;24:773. doi: 10.1186/s12864-023-09846-9 (PMC10720214; doi:10.1186/s12864-023-09846-9)
Supplement: Supplementary file 1 — Supplementary Material 1 [file 12864_2023_9846_MOESM1_ESM.docx]

**Supplementary Table 1:** A total of 347 Single Nucleotide Polymorphisms (SNPs) and the corresponding amino acid mutations in human receptor intercellular adhesion molecule-1 (ICAM-1).

| **S. no** | **dbSNP ID** | **Amino Acid substitution** | **If disordered in the 3D structure (PDB IDs: 5MZA, 6S8U)** | | **Domain** |
| --- | --- | --- | --- | --- | --- |
| 1 | [rs1366763648](https://www.ncbi.nlm.nih.gov/snp/rs1366763648) | M1V | disordered | |  |
|  | [rs1167645849](https://www.ncbi.nlm.nih.gov/snp/rs1167645849) | M1K | disordered | |  |
|  | [rs1055973323](https://www.ncbi.nlm.nih.gov/snp/rs1055973323) | M1I | disordered | |  |
| 2 | [rs758212257](https://www.ncbi.nlm.nih.gov/snp/rs758212257) | A2T | disordered | |  |
|  | [rs1255397267](https://www.ncbi.nlm.nih.gov/snp/rs1255397267) | A2V | disordered | |  |
| 3 | [rs779984305](https://www.ncbi.nlm.nih.gov/snp/rs779984305) | S4G | disordered | |  |
| 4 | [rs781040245](https://www.ncbi.nlm.nih.gov/snp/rs781040245) | P6S | disordered | |  |
| 5 | [rs747654731](https://www.ncbi.nlm.nih.gov/snp/rs747654731) | R7W | disordered | |  |
|  | [rs769191023](https://www.ncbi.nlm.nih.gov/snp/rs769191023) | R7P | disordered | |  |
| 6 | [rs1490803282](https://www.ncbi.nlm.nih.gov/snp/rs1490803282) | P8S | disordered | |  |
|  | [rs2039977212](https://www.ncbi.nlm.nih.gov/snp/rs2039977212) | P8L | disordered | |  |
| 7 | [rs1011691226](https://www.ncbi.nlm.nih.gov/snp/rs1011691226) | L10Q | disordered | |  |
| 8 | [rs770635391](https://www.ncbi.nlm.nih.gov/snp/rs770635391) | P11S | disordered | |  |
| 9 | [rs775798556](https://www.ncbi.nlm.nih.gov/snp/rs775798556) | A12S | disordered | |  |
|  | [rs764608366](https://www.ncbi.nlm.nih.gov/snp/rs764608366) | A12V | disordered | |  |
| 10 | [rs777039885](https://www.ncbi.nlm.nih.gov/snp/rs777039885) | L13F | disordered | |  |
| 11 | [rs1599262143](https://www.ncbi.nlm.nih.gov/snp/rs1599262143) | L14P | disordered | |  |
| 12 | [rs373461435](https://www.ncbi.nlm.nih.gov/snp/rs373461435) | V15I/L | disordered | |  |
| 13 | [rs750591481](https://www.ncbi.nlm.nih.gov/snp/rs750591481) | L17P | disordered | |  |
| 14 | [rs200158754](https://www.ncbi.nlm.nih.gov/snp/rs200158754) | G18R | disordered | |  |
| 15 | [rs2039977591](https://www.ncbi.nlm.nih.gov/snp/rs2039977591) | A19S | disordered | |  |
| 16 | [rs1017525934](https://www.ncbi.nlm.nih.gov/snp/rs1017525934) | L20V | disordered | |  |
| 17 | [rs1456986382](https://www.ncbi.nlm.nih.gov/snp/rs1456986382) | P22S | disordered | |  |
|  | [rs1213762840](https://www.ncbi.nlm.nih.gov/snp/rs1213762840) | P22L | disordered | |  |
| 18 | [rs759316086](https://www.ncbi.nlm.nih.gov/snp/rs759316086) | P24A | disordered | |  |
|  | [rs2040004926](https://www.ncbi.nlm.nih.gov/snp/rs2040004926) | P24L | disordered | |  |
| 19 | [rs2040004954](https://www.ncbi.nlm.nih.gov/snp/rs2040004954) | G25D | disordered | |  |
| 20 | [rs767201668](https://www.ncbi.nlm.nih.gov/snp/rs767201668) | A27T | disordered | |  |
| 21 | [rs1427679145](https://www.ncbi.nlm.nih.gov/snp/rs1427679145) | Q28R |  | | topological |
| 22 | [rs1353647708](https://www.ncbi.nlm.nih.gov/snp/rs1353647708) | T29I |  | | topological |
| 23 | [rs1227239485](https://www.ncbi.nlm.nih.gov/snp/rs1227239485) | S30F |  | | topological |
| 24 | [rs2040005097](https://www.ncbi.nlm.nih.gov/snp/rs2040005097) | S32T |  | | topological |
| 25 | [rs2145489444](https://www.ncbi.nlm.nih.gov/snp/rs2145489444) | P33S |  | | topological |
|  | [rs2040005114](https://www.ncbi.nlm.nih.gov/snp/rs2040005114) | P33L |  | | topological |
| 26 | [rs2145489451](https://www.ncbi.nlm.nih.gov/snp/rs2145489451) | S34P |  | | topological |
| 27 | [rs1214959993](https://www.ncbi.nlm.nih.gov/snp/rs1214959993) | K35T |  | | topological |
| 28 | [rs755971015](https://www.ncbi.nlm.nih.gov/snp/rs755971015) | V36I |  | | topological |
|  | [rs1486174155](https://www.ncbi.nlm.nih.gov/snp/rs1486174155) | V36G |  | | topological |
| 29 | [rs137977632](https://www.ncbi.nlm.nih.gov/snp/rs137977632) | P39S |  | | topological |
|  | [rs2040005253](https://www.ncbi.nlm.nih.gov/snp/rs2040005253) | P39L |  | | topological |
| 30 | [rs747111380](https://www.ncbi.nlm.nih.gov/snp/rs747111380) | R40W |  | | topological |
|  | rs756786973 | R40Q |  | | topological |
| 31 | [rs778474887](https://www.ncbi.nlm.nih.gov/snp/rs778474887) | G41R |  | | topological |
| 32 | rs771818150 | V44L |  | | topological |
| 33 | [rs2145489533](https://www.ncbi.nlm.nih.gov/snp/rs2145489533) | L45Q |  | | topological |
| 34 | [rs1435027936](https://www.ncbi.nlm.nih.gov/snp/rs1435027936) | C48S |  | | topological |
|  | [rs200156450](https://www.ncbi.nlm.nih.gov/snp/rs200156450) | C48W |  | | topological |
| 35 | [rs2040005531](https://www.ncbi.nlm.nih.gov/snp/rs2040005531) | T50A |  | | topological |
| 36 | [rs1485425363](https://www.ncbi.nlm.nih.gov/snp/rs1485425363) | S51Y |  | | topological |
| 37 | [rs773878586](https://www.ncbi.nlm.nih.gov/snp/rs773878586) | C52S |  | | topological |
| 38 | [rs763087198](https://www.ncbi.nlm.nih.gov/snp/rs763087198) | D53H |  | | topological |
|  | [rs1284791760](https://www.ncbi.nlm.nih.gov/snp/rs1284791760) | D53E |  | | topological |
| 39 | [rs200970471](https://www.ncbi.nlm.nih.gov/snp/rs200970471) | Q54R |  | | topological |
| 40 | [rs759672075](https://www.ncbi.nlm.nih.gov/snp/rs759672075) | P55A |  | | topological |
| 41 | [rs2145489661](https://www.ncbi.nlm.nih.gov/snp/rs2145489661) | K56E |  | | topological |
|  | [rs5491](https://www.ncbi.nlm.nih.gov/snp/rs5491) | K56R/M |  | | topological |
|  | [rs2145489695](https://www.ncbi.nlm.nih.gov/snp/rs2145489695) | K56N |  | | topological |
| 42 | [rs1183121001](https://www.ncbi.nlm.nih.gov/snp/rs1183121001) | T62A |  | | topological |
| 43 | [rs1468617162](https://www.ncbi.nlm.nih.gov/snp/rs1468617162) | P63S |  | | topological |
|  | [rs763975382](https://www.ncbi.nlm.nih.gov/snp/rs763975382) | P63L |  | | topological |
| 44 | [rs111997581](https://www.ncbi.nlm.nih.gov/snp/rs111997581) | L64M |  | | topological |
| 45 | [rs778384721](https://www.ncbi.nlm.nih.gov/snp/rs778384721) | E68D |  | | topological |
| 46 | [rs2040006105](https://www.ncbi.nlm.nih.gov/snp/rs2040006105) | L70F |  | | topological |
|  | [rs1400032879](https://www.ncbi.nlm.nih.gov/snp/rs1400032879) | L70P |  | | topological |
| 47 | [rs2145489753](https://www.ncbi.nlm.nih.gov/snp/rs2145489753) | P72R |  | | topological |
| 48 | rs374718798 | G73E |  | | topological |
| 49 | [rs369268780](https://www.ncbi.nlm.nih.gov/snp/rs369268780) | N74S |  | | topological |
|  | rs372704720 | N74K |  | | topological |
| 50 | CA403965495 (Clinical Allele Registry) | N75I |  | | topological |
| 51 | [rs146780348](https://www.ncbi.nlm.nih.gov/snp/rs1371251341) | R76W |  | | topological |
|  | rs554091291 | R76Q/L |  | |  |
| 52 | CA403965508 (Clinical Allele Registry) | K77N |  | |  |
| 53 | [rs1196902783](https://www.ncbi.nlm.nih.gov/snp/rs1196902783) | V78M |  | | topological |
|  | [rs774261335](https://www.ncbi.nlm.nih.gov/snp/rs774261335) | V78A |  | | topological |
| 54 | [rs2040006471](https://www.ncbi.nlm.nih.gov/snp/rs2040006471) | S82R |  | | topological |
| 55 | [rs1032707496](https://www.ncbi.nlm.nih.gov/snp/rs1032707496) | N83S |  | | topological |
|  | [rs1374469173](https://www.ncbi.nlm.nih.gov/snp/rs1374469173) | N83K |  | | topological |
| 56 | [rs746006779](https://www.ncbi.nlm.nih.gov/snp/rs746006779) | V84L |  | | topological |
| 57 | [rs2040006593](https://www.ncbi.nlm.nih.gov/snp/rs2040006593) | E86K |  | | topological |
| 58 | [rs1374649868](https://www.ncbi.nlm.nih.gov/snp/rs1374649868) | S88R |  | | topological |
| 59 | [rs772134103](https://www.ncbi.nlm.nih.gov/snp/rs772134103) | P90R |  | | topological |
| 60 | [rs2040006656](https://www.ncbi.nlm.nih.gov/snp/rs2040006656) | M91T |  | | topological |
|  | [rs140559142](https://www.ncbi.nlm.nih.gov/snp/rs140559142) | M91I |  | | topological |
| 61 | [rs1171324211](https://www.ncbi.nlm.nih.gov/snp/rs1171324211) | S94L |  | | topological |
| 62 | [rs914038707](https://www.ncbi.nlm.nih.gov/snp/rs914038707) | N95D |  | | topological |
|  | [rs2040006739](https://www.ncbi.nlm.nih.gov/snp/rs2040006739) | N95S |  | | topological |
| 63 | [rs1375741840](https://www.ncbi.nlm.nih.gov/snp/rs1375741840) | C96G |  | | topological |
| 64 | [rs542476616](https://www.ncbi.nlm.nih.gov/snp/rs542476616) | D98N/H |  | | topological |
| 65 | [rs1036900727](https://www.ncbi.nlm.nih.gov/snp/rs1036900727) | G99E |  | | topological |
| 66 | [rs2040006856](https://www.ncbi.nlm.nih.gov/snp/rs2040006856) | T102A |  | | topological |
| 67 | [rs765263284](https://www.ncbi.nlm.nih.gov/snp/rs765263284) | L107F/V |  | | topological |
| 68 | [rs561101249](https://www.ncbi.nlm.nih.gov/snp/rs561101249) | V109M |  | | topological |
| 69 | [rs2040006960](https://www.ncbi.nlm.nih.gov/snp/rs2040006960) | Y110H |  | | topological |
| 70 | [rs922664343](https://www.ncbi.nlm.nih.gov/snp/rs922664343) | W111R |  | | topological |
|  | [rs1171688556](https://www.ncbi.nlm.nih.gov/snp/rs1171688556) | W111L |  | | topological |
| 71 | [rs1294380638](https://www.ncbi.nlm.nih.gov/snp/rs1294380638) | T112S/A |  | | topological |
| 72 | [rs150480007](https://www.ncbi.nlm.nih.gov/snp/rs150480007) | R115W |  | | topological |
|  | [rs754203844](https://www.ncbi.nlm.nih.gov/snp/rs754203844) | R115Q |  | | topological |
| 73 | [rs757725637](https://www.ncbi.nlm.nih.gov/snp/rs757725637) | V116M |  | | topological |
| 74 | [rs1250014617](https://www.ncbi.nlm.nih.gov/snp/rs1250014617) | L118V |  | | topological |
| 75 | [rs1207079639](https://www.ncbi.nlm.nih.gov/snp/rs1207079639) | A119V |  | | topological |
| 76 | [rs1287926004](https://www.ncbi.nlm.nih.gov/snp/rs1287926004) | P120S |  | | topological |
| 77 | [rs1192864942](https://www.ncbi.nlm.nih.gov/snp/rs1192864942) | L121F |  | | topological |
| 78 | [rs750950257](https://www.ncbi.nlm.nih.gov/snp/rs750950257) | Q125R |  | | topological |
| 79 | [rs1013160528](https://www.ncbi.nlm.nih.gov/snp/rs1013160528) | V127M |  | | topological |
| 80 | [rs2040076382](https://www.ncbi.nlm.nih.gov/snp/rs2040076382) | G128C/S |  | | topological |
| 81 | [rs2145499149](https://www.ncbi.nlm.nih.gov/snp/rs2145499149) | N130D |  | | topological |
| 82 | [rs1204497162](https://www.ncbi.nlm.nih.gov/snp/rs1204497162) | L131H |  | | topological |
| 83 | [rs1454017997](https://www.ncbi.nlm.nih.gov/snp/rs1454017997) | T132I |  | | topological |
| 84 | [rs780182158](https://www.ncbi.nlm.nih.gov/snp/rs780182158) | L133V |  | | topological |
| 85 | [rs768846633](https://www.ncbi.nlm.nih.gov/snp/rs768846633) | R134C/G/S |  | | topological |
|  | [rs781416568](https://www.ncbi.nlm.nih.gov/snp/rs781416568) | R134L/H |  | | topological |
| 86 | [rs2040076672](https://www.ncbi.nlm.nih.gov/snp/rs2040076672) | C135F |  | | topological |
| 87 | [rs769636060](https://www.ncbi.nlm.nih.gov/snp/rs769636060) | V137M |  | | topological |
|  | [rs1405414298](https://www.ncbi.nlm.nih.gov/snp/rs1405414298) | V137A |  | | topological |
| 88 | [rs1349831256](https://www.ncbi.nlm.nih.gov/snp/rs1349831256) | G139S |  | | topological |
|  | [rs377028532](https://www.ncbi.nlm.nih.gov/snp/rs377028532) | G139D |  | | topological |
| 89 | [rs773974462](https://www.ncbi.nlm.nih.gov/snp/rs773974462) | R143W |  | | topological |
|  | [rs373279831](https://www.ncbi.nlm.nih.gov/snp/rs373279831) | R143P |  | | topological |
|  |  | R143Q |  | |  |
| 90 | [rs2040077178](https://www.ncbi.nlm.nih.gov/snp/rs2040077178) | N145D |  | | topological |
| 91 | [rs1351321520](https://www.ncbi.nlm.nih.gov/snp/rs1351321520) | T147P |  | | topological |
|  | [rs1408852700](https://www.ncbi.nlm.nih.gov/snp/rs1408852700) | T147S |  | | topological |
| 92 | [rs201079272](https://www.ncbi.nlm.nih.gov/snp/rs201079272) | V148M |  | | topological |
| 93 | [rs1599270102](https://www.ncbi.nlm.nih.gov/snp/rs1599270102) | L150Q |  | | topological |
| 94 | [rs765617669](https://www.ncbi.nlm.nih.gov/snp/rs765617669) | L151V |  | | topological |
| 95 | [rs750824008](https://www.ncbi.nlm.nih.gov/snp/rs750824008) | R152C |  | | topological |
|  | [rs370720409](https://www.ncbi.nlm.nih.gov/snp/rs370720409) | R152L/H |  | | topological |
| 96 | [rs2040077620](https://www.ncbi.nlm.nih.gov/snp/rs2040077620) | G153E |  | | topological |
| 97 | [rs1378091078](https://www.ncbi.nlm.nih.gov/snp/rs1378091078) | K155E |  | | topological |
|  | [rs5492](https://www.ncbi.nlm.nih.gov/snp/rs5492) | K155N |  | | topological |
| 98 | [rs2040077776](https://www.ncbi.nlm.nih.gov/snp/rs2040077776) | E156D |  | | topological |
| 99 | [rs2040077808](https://www.ncbi.nlm.nih.gov/snp/rs2040077808) | K158R |  | | topological |
| 100 | [rs375107327](https://www.ncbi.nlm.nih.gov/snp/rs375107327) | R159W |  | | topological |
|  | [rs1361221966](https://www.ncbi.nlm.nih.gov/snp/rs1361221966) | R159L/Q |  | | topological |
| 101 | [rs2145499295](https://www.ncbi.nlm.nih.gov/snp/rs2145499295) | P161L |  | | topological |
| 102 | [rs367947901](https://www.ncbi.nlm.nih.gov/snp/rs367947901) | A162D |  | | topological |
| 103 | [rs1465004002](https://www.ncbi.nlm.nih.gov/snp/rs1465004002) | V163L/M |  | | topological |
| 104 | [rs1198112599](https://www.ncbi.nlm.nih.gov/snp/rs1198112599) | G164A |  | | topological |
| 105 | [rs1307423569](https://www.ncbi.nlm.nih.gov/snp/rs1307423569) | E165K |  | | topological |
| 106 | [rs2145499329](https://www.ncbi.nlm.nih.gov/snp/rs2145499329) | P166S |  | | topological |
|  | [rs2040078232](https://www.ncbi.nlm.nih.gov/snp/rs2040078232) | P166H |  | | topological |
| 107 | [rs777593675](https://www.ncbi.nlm.nih.gov/snp/rs777593675) | A167G |  | | topological |
|  | rs769975581 | A167T |  | |  |
| 108 | [rs143328428](https://www.ncbi.nlm.nih.gov/snp/rs143328428) | E168K |  | | topological |
|  | [rs749083718](https://www.ncbi.nlm.nih.gov/snp/rs749083718) | E168A |  | | topological |
| 109 | [rs770851229](https://www.ncbi.nlm.nih.gov/snp/rs770851229) | T170R/M |  | | topological |
| 110 | [rs759046761](https://www.ncbi.nlm.nih.gov/snp/rs759046761) | T172R/M |  | | topological |
| 111 | [rs924525389](https://www.ncbi.nlm.nih.gov/snp/rs924525389) | V173M |  | | topological |
| 112 | [rs1431740611](https://www.ncbi.nlm.nih.gov/snp/rs1431740611) | V175M |  | | topological |
| 113 | [rs2145499397](https://www.ncbi.nlm.nih.gov/snp/rs2145499397) | R176G |  | | topological |
|  | [rs934593597](https://www.ncbi.nlm.nih.gov/snp/rs934593597) | R176S |  | | topological |
| 114 | [rs771716527](https://www.ncbi.nlm.nih.gov/snp/rs771716527) | D178N |  | | topological |
| 115 | [rs1051567834](https://www.ncbi.nlm.nih.gov/snp/rs1051567834) | H179N/Y |  | | topological |
| 116 | [rs139263745](https://www.ncbi.nlm.nih.gov/snp/rs139263745) | H180R |  | | topological |
| 117 | [rs954408067](https://www.ncbi.nlm.nih.gov/snp/rs954408067) | A182G |  | | topological |
| 118 | [rs2040079023](https://www.ncbi.nlm.nih.gov/snp/rs2040079023) | N183D |  | | topological |
|  | [rs760374267](https://www.ncbi.nlm.nih.gov/snp/rs760374267) | N183S |  | | topological |
| 119 | [rs1441940436](https://www.ncbi.nlm.nih.gov/snp/rs1441940436) | S185L |  | | topological |
| 120 | [rs1007384990](https://www.ncbi.nlm.nih.gov/snp/rs1007384990) | R187C/G/S |  | | topological |
|  | [rs766833990](https://www.ncbi.nlm.nih.gov/snp/rs766833990) | R187L/H |  | | topological |
| 121 | [rs1300574941](https://www.ncbi.nlm.nih.gov/snp/rs1300574941) | T188A |  | | topological |
| 122 | [rs767572203](https://www.ncbi.nlm.nih.gov/snp/rs767572203) | E189A |  | | topological |
| 123 | [rs139053442](https://www.ncbi.nlm.nih.gov/snp/rs139053442) | D191H |  | | topological |
| 124 | [rs372112882](https://www.ncbi.nlm.nih.gov/snp/rs372112882) | R193W |  | | topological |
|  | rs777879275 | R193Q |  | | topological |
| 125 | [rs757120426](https://www.ncbi.nlm.nih.gov/snp/rs757120426) | P194S/A |  | | topological |
| 126 | [rs137905196](https://www.ncbi.nlm.nih.gov/snp/rs137905196) | G196E |  | | topological |
| 127 | [rs2040079656](https://www.ncbi.nlm.nih.gov/snp/rs2040079656) | E198K/Q |  | | topological |
| 128 | [rs1053511706](https://www.ncbi.nlm.nih.gov/snp/rs1053511706) | E201K |  | | topological |
| 129 | [rs775112898](https://www.ncbi.nlm.nih.gov/snp/rs775112898) | N202K |  | | topological |
| 130 | [rs1434090171](https://www.ncbi.nlm.nih.gov/snp/rs1434090171) | T203S/P |  | | topological |
| 131 | [rs746554151](https://www.ncbi.nlm.nih.gov/snp/rs746554151) | S204L |  | | topological |
| 132 | [rs373913806](https://www.ncbi.nlm.nih.gov/snp/rs373913806) | A205T |  | | topological |
|  |  | A205V |  | |  |
| 133 | [rs1045821147](https://www.ncbi.nlm.nih.gov/snp/rs1045821147) | P206T |  | | topological |
|  | [rs1339364571](https://www.ncbi.nlm.nih.gov/snp/rs1339364571) | P206H |  | | topological |
| 134 | [rs2145499589](https://www.ncbi.nlm.nih.gov/snp/rs2145499589) | Y207H |  | | topological |
|  | [rs766744082](https://www.ncbi.nlm.nih.gov/snp/rs766744082) | Y207C/F |  | | topological |
| 135 | [rs1220318523](https://www.ncbi.nlm.nih.gov/snp/rs1220318523) | Q210R |  | | topological |
|  | [rs141554198](https://www.ncbi.nlm.nih.gov/snp/rs141554198) | Q210H |  | | topological |
| 136 | [rs2040080157](https://www.ncbi.nlm.nih.gov/snp/rs2040080157) | F212S |  | |  |
| 137 | [rs1599270516](https://www.ncbi.nlm.nih.gov/snp/rs1599270516) | V213A | disordered | |  |
| 138 | [rs1441369918](https://www.ncbi.nlm.nih.gov/snp/rs1441369918) | A216T | disordered | |  |
|  | [rs747745154](https://www.ncbi.nlm.nih.gov/snp/rs747745154) | A216V/E | disordered | |  |
| 139 | rs1414989542 | T217A | disordered | |  |
| 140 | [rs2040082799](https://www.ncbi.nlm.nih.gov/snp/rs2040082799) | P218S | disordered | |  |
|  | [rs150484122](https://www.ncbi.nlm.nih.gov/snp/rs150484122) | P218L | disordered | |  |
| 141 | [rs1446643875](https://www.ncbi.nlm.nih.gov/snp/rs1446643875) | P219T | disordered | |  |
|  | rs201414539 | P219R/Q | disordered | |  |
| 142 | [rs200325260](https://www.ncbi.nlm.nih.gov/snp/rs200325260) | Q220R | disordered | |  |
| 143 | [rs760747058](https://www.ncbi.nlm.nih.gov/snp/rs760747058) | L221I | disordered | |  |
| 144 | [rs1238479170](https://www.ncbi.nlm.nih.gov/snp/rs1238479170) | V222F | disordered | |  |
|  | [rs556179599](https://www.ncbi.nlm.nih.gov/snp/rs556179599) | V222G | disordered | |  |
| 145 | [rs1221439292](https://www.ncbi.nlm.nih.gov/snp/rs1221439292) | P224A | disordered | |  |
| 146 | [rs1568295295](https://www.ncbi.nlm.nih.gov/snp/rs1568295295) | R225W | disordered | |  |
|  | [rs754014530](https://www.ncbi.nlm.nih.gov/snp/rs754014530) | R225Q/P | disordered | |  |
| 147 | [rs1199329249](https://www.ncbi.nlm.nih.gov/snp/rs1199329249) | V226L/I | disordered | |  |
| 148 | [rs1337841278](https://www.ncbi.nlm.nih.gov/snp/rs1337841278) | E228K | disordered | |  |
| 149 | [rs765607464](https://www.ncbi.nlm.nih.gov/snp/rs765607464) | V229M | disordered | |  |
| 150 | [rs139607484](https://www.ncbi.nlm.nih.gov/snp/rs139607484) | D230N | disordered | |  |
| 151 | [rs1379464546](https://www.ncbi.nlm.nih.gov/snp/rs1379464546) | T231A | disordered | |  |
|  | [rs375023616](https://www.ncbi.nlm.nih.gov/snp/rs375023616) | T231M | disordered | |  |
| 152 | [rs755002696](https://www.ncbi.nlm.nih.gov/snp/rs755002696) | G233R | disordered | |  |
|  | [rs1413458415](https://www.ncbi.nlm.nih.gov/snp/rs1413458415) | G233E | disordered | |  |
| 153 | [rs769386131](https://www.ncbi.nlm.nih.gov/snp/rs769386131) | V235M | disordered | |  |
|  | [rs2040083422](https://www.ncbi.nlm.nih.gov/snp/rs2040083422) | V235A | disordered | |  |
| 154 | [rs777434435](https://www.ncbi.nlm.nih.gov/snp/rs777434435) | V236F | disordered | |  |
|  | [rs534458967](https://www.ncbi.nlm.nih.gov/snp/rs534458967) | V236A | disordered | |  |
| 155 | [rs1599270631](https://www.ncbi.nlm.nih.gov/snp/rs1599270631) | C237S | disordered | |  |
| 156 | [rs772332794](https://www.ncbi.nlm.nih.gov/snp/rs772332794) | S238Y/F | disordered | |  |
| 157 | [rs1182296722](https://www.ncbi.nlm.nih.gov/snp/rs1182296722) | L239P | disordered | |  |
| 158 | [rs761079910](https://www.ncbi.nlm.nih.gov/snp/rs761079910) | D240A | disordered | |  |
| 159 | [rs1799969](https://www.ncbi.nlm.nih.gov/snp/rs1799969) | G241R | disordered | |  |
| 160 | [rs1005199205](https://www.ncbi.nlm.nih.gov/snp/rs1005199205) | L242V | disordered | |  |
| 161 | [rs369391699](https://www.ncbi.nlm.nih.gov/snp/rs369391699) | F243L | disordered | |  |
| 162 | rs2040083799 | P244Q | disordered | |  |
| 163 | [rs765520096](https://www.ncbi.nlm.nih.gov/snp/rs765520096) | S246L | disordered | |  |
| 164 | [rs2040083897](https://www.ncbi.nlm.nih.gov/snp/rs2040083897) | A248T | disordered | |  |
| 165 | rs2040083916 | H251Y | disordered | |  |
|  | [rs2040083936](https://www.ncbi.nlm.nih.gov/snp/rs2040083936) | H251L | disordered | |  |
|  | [rs930526340](https://www.ncbi.nlm.nih.gov/snp/rs930526340) | H251Q | disordered | |  |
| 166 | [rs762780304](https://www.ncbi.nlm.nih.gov/snp/rs762780304) | L252P | disordered | |  |
| 167 | rs139628843 | Q257H | disordered | |  |
| 168 | [rs1599270705](https://www.ncbi.nlm.nih.gov/snp/rs1599270705) | R258G | disordered | |  |
|  | [rs142745312](https://www.ncbi.nlm.nih.gov/snp/rs142745312) | R258K | disordered | |  |
| 169 | [rs781210616](https://www.ncbi.nlm.nih.gov/snp/rs781210616) | P261T | disordered | |  |
| 170 | rs907641822 | T262P/S | disordered | |  |
| 171 | [rs752161214](https://www.ncbi.nlm.nih.gov/snp/rs752161214) | Y265S | disordered | |  |
| 172 | [rs755625816](https://www.ncbi.nlm.nih.gov/snp/rs755625816) | N267T | disordered | |  |
| 173 | [rs748922559](https://www.ncbi.nlm.nih.gov/snp/rs748922559) | D268N | disordered | |  |
| 174 | [rs2040084309](https://www.ncbi.nlm.nih.gov/snp/rs2040084309) | S269P | disordered | |  |
|  | [rs2040084337](https://www.ncbi.nlm.nih.gov/snp/rs2040084337) | S269F | disordered | |  |
| 175 | [rs780248758](https://www.ncbi.nlm.nih.gov/snp/rs780248758) | F270S | disordered | |  |
|  | [rs34369517](https://www.ncbi.nlm.nih.gov/snp/rs34369517) | F270L | disordered | |  |
| 176 | [rs747446910](https://www.ncbi.nlm.nih.gov/snp/rs747446910) | S271L | disordered | |  |
| 177 | [rs1266308413](https://www.ncbi.nlm.nih.gov/snp/rs1266308413) | A272S | disordered | |  |
| 178 | [rs567512435](https://www.ncbi.nlm.nih.gov/snp/rs567512435) | A274T/P | disordered | |  |
|  | [rs770010762](https://www.ncbi.nlm.nih.gov/snp/rs770010762) | A274V | disordered | |  |
| 179 | [rs2145500177](https://www.ncbi.nlm.nih.gov/snp/rs2145500177) | S277R/C | disordered | |  |
| 180 | [rs535206172](https://www.ncbi.nlm.nih.gov/snp/rs535206172) | V278M | disordered | |  |
|  | [rs763265816](https://www.ncbi.nlm.nih.gov/snp/rs763265816) | V278G | disordered | |  |
| 181 | [rs141080585](https://www.ncbi.nlm.nih.gov/snp/rs141080585) | A280T/S | disordered | |  |
| 182 | [rs1368501868](https://www.ncbi.nlm.nih.gov/snp/rs1368501868) | D282N | disordered | |  |
|  | rs1000391834 | D282G | disordered | |  |
| 183 | [rs113419367](https://www.ncbi.nlm.nih.gov/snp/rs113419367) | E283K | disordered | |  |
|  | [rs2040084833](https://www.ncbi.nlm.nih.gov/snp/rs2040084833) | E283D | disordered | |  |
| 184 | [rs2040084850](https://www.ncbi.nlm.nih.gov/snp/rs2040084850) | G284S | disordered | |  |
|  | [rs2040084867](https://www.ncbi.nlm.nih.gov/snp/rs2040084867) | G284D | disordered | |  |
| 185 | [rs2040084889](https://www.ncbi.nlm.nih.gov/snp/rs2040084889) | T285A | disordered | |  |
|  | [rs1294424687](https://www.ncbi.nlm.nih.gov/snp/rs1294424687) | T285I | disordered | |  |
| 186 | [rs756098611](https://www.ncbi.nlm.nih.gov/snp/rs756098611) | Q286H | disordered | |  |
| 187 | [rs989473316](https://www.ncbi.nlm.nih.gov/snp/rs989473316) | R287W | disordered | |  |
|  | [rs763586600](https://www.ncbi.nlm.nih.gov/snp/rs763586600) | R287Q | disordered | |  |
| 188 | [rs964564309](https://www.ncbi.nlm.nih.gov/snp/rs964564309) | L288Q | disordered | |  |
| 189 | [rs2040085060](https://www.ncbi.nlm.nih.gov/snp/rs2040085060) | T289A | disordered | |  |
|  | [rs147925015](https://www.ncbi.nlm.nih.gov/snp/rs147925015) | T289R/M | disordered | |  |
| 190 | [rs1342293195](https://www.ncbi.nlm.nih.gov/snp/rs1342293195) | L294P | disordered | |  |
| 191 | [rs2040085198](https://www.ncbi.nlm.nih.gov/snp/rs2040085198) | G295R | disordered | |  |
|  | [rs755378643](https://www.ncbi.nlm.nih.gov/snp/rs755378643) | G295V/E | disordered | |  |
| 192 | [rs1404271908](https://www.ncbi.nlm.nih.gov/snp/rs1404271908) | N296S | disordered | |  |
|  | [rs2145500276](https://www.ncbi.nlm.nih.gov/snp/rs2145500276) | N296K | disordered | |  |
| 193 | [rs369906592](https://www.ncbi.nlm.nih.gov/snp/rs369906592) | S298N | disordered | |  |
| 194 | [rs2145500283](https://www.ncbi.nlm.nih.gov/snp/rs2145500283) | Q299R | disordered | |  |
| 195 | [rs2040085299](https://www.ncbi.nlm.nih.gov/snp/rs2040085299) | T301A/S | disordered | |  |
|  | [rs2040085329](https://www.ncbi.nlm.nih.gov/snp/rs2040085329) | T301R | disordered | |  |
| 196 | [rs2040085346](https://www.ncbi.nlm.nih.gov/snp/rs2040085346) | L302M | disordered | |  |
| 197 | [rs773397826](https://www.ncbi.nlm.nih.gov/snp/rs773397826) | T304R | disordered | |  |
| 198 | [rs1424977643](https://www.ncbi.nlm.nih.gov/snp/rs1424977643) | I307V | disordered | |  |
| 199 | [rs1185042029](https://www.ncbi.nlm.nih.gov/snp/rs1185042029) | Y308H | disordered | |  |
| 200 | [rs1384202081](https://www.ncbi.nlm.nih.gov/snp/rs1384202081) | S309G | disordered | |  |
| 201 | rs1401064916 | P311S | disordered | |  |
|  | [rs772315973](https://www.ncbi.nlm.nih.gov/snp/rs772315973) | P311L | disordered | |  |
| 202 | [rs768543917](https://www.ncbi.nlm.nih.gov/snp/rs768543917) | A312E/V | disordered | |  |
| 203 | [rs764794802](https://www.ncbi.nlm.nih.gov/snp/rs764794802) | P313S/A | disordered | |  |
| 204 | [rs1411318260](https://www.ncbi.nlm.nih.gov/snp/rs1411318260) | P313R | disordered | |  |
| 205 | [rs2040086602](https://www.ncbi.nlm.nih.gov/snp/rs2040086602) | N314D | disordered | |  |
|  | rs762664096 | N314S/I | disordered | |  |
| 206 | [rs5495](https://www.ncbi.nlm.nih.gov/snp/rs5495) | V315M | disordered | |  |
| 207 | [rs756550665](https://www.ncbi.nlm.nih.gov/snp/rs756550665) | L317Q | disordered | |  |
| 208 | [rs1235964865](https://www.ncbi.nlm.nih.gov/snp/rs1235964865) | T318M | disordered | |  |
| 209 | [rs1192108985](https://www.ncbi.nlm.nih.gov/snp/rs1192108985) | P320S | disordered | |  |
| 210 | [rs1269866645](https://www.ncbi.nlm.nih.gov/snp/rs1269866645) | E321G/V | disordered | |  |
| 211 | [rs754356021](https://www.ncbi.nlm.nih.gov/snp/rs754356021) | V322F | disordered | |  |
| 212 | [rs1431972317](https://www.ncbi.nlm.nih.gov/snp/rs1431972317) | S323L | disordered | |  |
| 213 | [rs368283911](https://www.ncbi.nlm.nih.gov/snp/rs368283911) | E324D | disordered | |  |
| 214 | rs1008944257 | T326N | disordered | |  |
| 215 | [rs143008699](https://www.ncbi.nlm.nih.gov/snp/rs143008699) | E327K/Q | disordered | |  |
|  | [rs746741810](https://www.ncbi.nlm.nih.gov/snp/rs746741810) | E327A | disordered | |  |
| 216 | [rs146134321](https://www.ncbi.nlm.nih.gov/snp/rs146134321) | V330M | disordered | |  |
|  | [rs1325801458](https://www.ncbi.nlm.nih.gov/snp/rs1325801458) | V330A | disordered | |  |
| 217 | [rs964678962](https://www.ncbi.nlm.nih.gov/snp/rs964678962) | K331T | disordered | |  |
| 218 | [rs769659570](https://www.ncbi.nlm.nih.gov/snp/rs769659570) | C332Y | disordered | |  |
| 219 | rs772757657 | P336A/S | disordered | |  |
|  | rs1285113750 | P336R | disordered | |  |
| 220 | [rs762576033](https://www.ncbi.nlm.nih.gov/snp/rs762576033) | R337T | disordered | |  |
| 221 | [rs960494429](https://www.ncbi.nlm.nih.gov/snp/rs960494429) | A338S | disordered | |  |
|  | [rs1340666091](https://www.ncbi.nlm.nih.gov/snp/rs1340666091) |  | |  |  |
| 222 | [rs141326678](https://www.ncbi.nlm.nih.gov/snp/rs141326678) | T341R/M | disordered | |  |
| 223 | [rs2040087318](https://www.ncbi.nlm.nih.gov/snp/rs2040087318) | N343I | disordered | |  |
|  | [rs754190930](https://www.ncbi.nlm.nih.gov/snp/rs754190930) | N343K | disordered | |  |
| 224 | [rs2040087361](https://www.ncbi.nlm.nih.gov/snp/rs2040087361) | G344R | disordered | |  |
|  | [rs1568295575](https://www.ncbi.nlm.nih.gov/snp/rs1568295575) | G344E | disordered | |  |
| 225 | [rs779307629](https://www.ncbi.nlm.nih.gov/snp/rs779307629) | V345I/L | disordered | |  |
|  | [rs1424635602](https://www.ncbi.nlm.nih.gov/snp/rs1424635602) | L345M/V |  | |  |
| 226 | rs780195275 | P346S | disordered | |  |
|  | [rs747223390](https://www.ncbi.nlm.nih.gov/snp/rs747223390) | P346L | disordered | |  |
| 227 | [rs768938327](https://www.ncbi.nlm.nih.gov/snp/rs768938327) | A347P/T | disordered | |  |
|  | [rs1027972658](https://www.ncbi.nlm.nih.gov/snp/rs1027972658) | A347V | disordered | |  |
| 228 | [rs2040087692](https://www.ncbi.nlm.nih.gov/snp/rs2040087692) | Q348E | disordered | |  |
|  | [rs2040087711](https://www.ncbi.nlm.nih.gov/snp/rs2040087711) | Q348H | disordered | |  |
| 229 | [rs983667097](https://www.ncbi.nlm.nih.gov/snp/rs983667097) | L350R | disordered | |  |
| 230 | rs1167107227 | P352A | disordered | |  |
|  | rs1801714 | P352L | disordered | |  |
| 231 | [rs1406188712](https://www.ncbi.nlm.nih.gov/snp/rs1406188712) | R353G | disordered | |  |
| 232 | [rs2145500669](https://www.ncbi.nlm.nih.gov/snp/rs2145500669) | A354V | disordered | |  |
| 233 | [rs868375367](https://www.ncbi.nlm.nih.gov/snp/rs868375367) | Q355H | disordered | |  |
| 234 | [rs2040087877](https://www.ncbi.nlm.nih.gov/snp/rs2040087877) | L356V/F | disordered | |  |
| 235 | [rs1302535118](https://www.ncbi.nlm.nih.gov/snp/rs1302535118) | A360P | disordered | |  |
| 236 | [rs1568295632](https://www.ncbi.nlm.nih.gov/snp/rs1568295632) | T361I | disordered | |  |
| 237 | [rs770551007](https://www.ncbi.nlm.nih.gov/snp/rs770551007) | P362T/S | disordered | |  |
| 238 | [rs199672020](https://www.ncbi.nlm.nih.gov/snp/rs199672020) | E363Q | disordered | |  |
|  | [rs1267923581](https://www.ncbi.nlm.nih.gov/snp/rs1267923581) | E363D | disordered | |  |
| 239 | [rs370415118](https://www.ncbi.nlm.nih.gov/snp/rs370415118) | G366R | disordered | |  |
| 240 | [rs139178890](https://www.ncbi.nlm.nih.gov/snp/rs139178890) | R367G/C | disordered | |  |
|  | [rs146000551](https://www.ncbi.nlm.nih.gov/snp/rs146000551) | R367H/L | disordered | |  |
| 241 | [rs139290643](https://www.ncbi.nlm.nih.gov/snp/rs139290643) | F369L | disordered | |  |
| 242 | [rs1489572878](https://www.ncbi.nlm.nih.gov/snp/rs1489572878) | S370F | disordered | |  |
| 243 | [rs2145500742](https://www.ncbi.nlm.nih.gov/snp/rs2145500742) | C371R | disordered | |  |
| 244 | [rs758965302](https://www.ncbi.nlm.nih.gov/snp/rs758965302) | S372F | disordered | |  |
| 245 | [rs1241607771](https://www.ncbi.nlm.nih.gov/snp/rs1241607771) | A373V | disordered | |  |
| 246 | [rs1483579827](https://www.ncbi.nlm.nih.gov/snp/rs1483579827) | T374I | disordered | |  |
| 247 | [rs2040088417](https://www.ncbi.nlm.nih.gov/snp/rs2040088417) | E376V | disordered | |  |
| 248 | [rs766445571](https://www.ncbi.nlm.nih.gov/snp/rs766445571) | V377M/L | disordered | |  |
| 249 | [rs2040088507](https://www.ncbi.nlm.nih.gov/snp/rs2040088507) | A378T | disordered | |  |
|  | [rs755206701](https://www.ncbi.nlm.nih.gov/snp/rs755206701) | A378V | disordered | |  |
| 250 | [rs376622574](https://www.ncbi.nlm.nih.gov/snp/rs376622574) | G379S/R | disordered | |  |
|  | [rs1380629463](https://www.ncbi.nlm.nih.gov/snp/rs1380629463) | G379D | disordered | |  |
| 251 | rs2040088670 | L381V | disordered | |  |
| 252 | [rs1036180125](https://www.ncbi.nlm.nih.gov/snp/rs1036180125) | I382T | disordered | |  |
| 253 | rs777679809 | H383P/R | disordered | |  |
| 254 | [rs1792839565](https://www.ncbi.nlm.nih.gov/snp/rs1792839565) | K384E | disordered | |  |
| 255 | [rs1599271274](https://www.ncbi.nlm.nih.gov/snp/rs1599271274) | T387I | disordered | |  |
| 256 | [rs181563357](https://www.ncbi.nlm.nih.gov/snp/rs181563357) | R388W | disordered | |  |
|  | [rs770432918](https://www.ncbi.nlm.nih.gov/snp/rs770432918) | R388Q | disordered | |  |
| 257 | [rs2040088934](https://www.ncbi.nlm.nih.gov/snp/rs2040088934) | E389G | disordered | |  |
|  | [rs1244743412](https://www.ncbi.nlm.nih.gov/snp/rs1244743412) | E389D | disordered | |  |
| 258 | rs5497 | R397Q | disordered | |  |
| 259 | [rs201464213](https://www.ncbi.nlm.nih.gov/snp/rs201464213) | E400K | disordered | |  |
| 260 | rs752861754 | R401T | disordered | |  |
| 261 | [rs760785172](https://www.ncbi.nlm.nih.gov/snp/rs760785172) | D402N | disordered | |  |
|  | [rs147967899](https://www.ncbi.nlm.nih.gov/snp/rs147967899) | D402G | disordered | |  |
|  | [rs2040090462](https://www.ncbi.nlm.nih.gov/snp/rs2040090462) | D402E | disordered | |  |
| 262 | [rs112301533](https://www.ncbi.nlm.nih.gov/snp/rs112301533) | P404R/L | disordered | |  |
| 263 | [rs1599271465](https://www.ncbi.nlm.nih.gov/snp/rs1599271465) | G405R | disordered | |  |
|  | [rs757120819](https://www.ncbi.nlm.nih.gov/snp/rs757120819) | G405A | disordered | |  |
| 264 | [rs368566175](https://www.ncbi.nlm.nih.gov/snp/rs368566175) | N406S | disordered | |  |
| 265 | [rs2040090578](https://www.ncbi.nlm.nih.gov/snp/rs2040090578) | W407R | disordered | |  |
|  | [rs971926313](https://www.ncbi.nlm.nih.gov/snp/rs971926313) | W407L | disordered | |  |
| 266 | [rs1281922240](https://www.ncbi.nlm.nih.gov/snp/rs1281922240) | T408P | disordered | |  |
|  | [rs371463487](https://www.ncbi.nlm.nih.gov/snp/rs371463487) | T408M | disordered | |  |
| 267 | [rs2145501060](https://www.ncbi.nlm.nih.gov/snp/rs2145501060) | P410S | disordered | |  |
| 268 | [rs935025840](https://www.ncbi.nlm.nih.gov/snp/rs935025840) | S413P | disordered | |  |
|  | [rs758430171](https://www.ncbi.nlm.nih.gov/snp/rs758430171) | S413C | disordered | |  |
| 269 | [rs199931094](https://www.ncbi.nlm.nih.gov/snp/rs199931094) | Q414H | disordered | |  |
| 270 | [rs2040090793](https://www.ncbi.nlm.nih.gov/snp/rs2040090793) | T416A | disordered | |  |
| 271 | [rs768253249](https://www.ncbi.nlm.nih.gov/snp/rs768253249) | M418V | disordered | |  |
|  | [rs2040090861](https://www.ncbi.nlm.nih.gov/snp/rs2040090861) | M418T | disordered | |  |
| 272 | [rs2040090898](https://www.ncbi.nlm.nih.gov/snp/rs2040090898) | A421S | disordered | |  |
|  | [rs1266669206](https://www.ncbi.nlm.nih.gov/snp/rs1266669206) | A421D | disordered | |  |
| 273 | [rs905083025](https://www.ncbi.nlm.nih.gov/snp/rs905083025) | W422S | disordered | |  |
| 274 | [rs1479852844](https://www.ncbi.nlm.nih.gov/snp/rs1479852844) | G423R | disordered | |  |
| 275 | [rs552634203](https://www.ncbi.nlm.nih.gov/snp/rs552634203) | N424I | disordered | |  |
| 276 | [rs1568295825](https://www.ncbi.nlm.nih.gov/snp/rs1568295825) | L426S | disordered | |  |
|  | rs2040091091 | L426F | disordered | |  |
| 277 | [rs2145501131](https://www.ncbi.nlm.nih.gov/snp/rs2145501131) | P427L | disordered | |  |
| 278 | [rs147723291](https://www.ncbi.nlm.nih.gov/snp/rs147723291) | E428K | disordered | |  |
|  | [rs2040091182](https://www.ncbi.nlm.nih.gov/snp/rs2040091182) | E428G/V | disordered | |  |
|  | [rs1391985429](https://www.ncbi.nlm.nih.gov/snp/rs1391985429) | E428D | disordered | |  |
| 279 | rs2040091227 | K430R | disordered | |  |
|  | [rs759927316](https://www.ncbi.nlm.nih.gov/snp/rs759927316) | K430N | disordered | |  |
| 280 | [rs2040091256](https://www.ncbi.nlm.nih.gov/snp/rs2040091256) | C431Y | disordered | |  |
| 281 | [rs2040091284](https://www.ncbi.nlm.nih.gov/snp/rs2040091284) | L432Q | disordered | |  |
| 282 | [rs772680139](https://www.ncbi.nlm.nih.gov/snp/rs772680139) | T436S/I | disordered | |  |
| 283 | [rs1401097415](https://www.ncbi.nlm.nih.gov/snp/rs1401097415) | I441V | disordered | |  |
| 284 | [rs374722531](https://www.ncbi.nlm.nih.gov/snp/rs374722531) | G442R | disordered | |  |
| 285 | [rs867279367](https://www.ncbi.nlm.nih.gov/snp/rs867279367) | E443K | disordered | |  |
|  | rs148613735 | E443G | disordered | |  |
| 286 | [rs1285002056](https://www.ncbi.nlm.nih.gov/snp/rs1285002056) | S444L | disordered | |  |
| 287 | [rs1356862171](https://www.ncbi.nlm.nih.gov/snp/rs1356862171) | V445L | disordered | |  |
| 288 | [rs140768360](https://www.ncbi.nlm.nih.gov/snp/rs140768360) | T446A | disordered | |  |
| 289 | [rs1599271567](https://www.ncbi.nlm.nih.gov/snp/rs1599271567) | T448P | disordered | |  |
| 290 | [rs1490175275](https://www.ncbi.nlm.nih.gov/snp/rs1490175275) | R449G | disordered | |  |
|  | [rs13306430](https://www.ncbi.nlm.nih.gov/snp/rs13306430) | R449Q | disordered | |  |
| 291 | [rs753651400](https://www.ncbi.nlm.nih.gov/snp/rs753651400) | D450N | disordered | |  |
|  | [rs199727082](https://www.ncbi.nlm.nih.gov/snp/rs199727082) | D450E | disordered | |  |
| 292 | [rs2040091810](https://www.ncbi.nlm.nih.gov/snp/rs2040091810) | G453S | disordered | |  |
| 293 | [rs1446861335](https://www.ncbi.nlm.nih.gov/snp/rs1446861335) | T454P | disordered | |  |
|  | [rs754492552](https://www.ncbi.nlm.nih.gov/snp/rs754492552) | T454I | disordered | |  |
| 294 | [rs780768999](https://www.ncbi.nlm.nih.gov/snp/rs780768999) | L456V | disordered | |  |
| 295 | [rs747787849](https://www.ncbi.nlm.nih.gov/snp/rs747787849) | C457Y/F | disordered | |  |
| 296 | [rs150121537](https://www.ncbi.nlm.nih.gov/snp/rs150121537) | R458W | disordered | |  |
|  | [rs779121366](https://www.ncbi.nlm.nih.gov/snp/rs779121366) | R458Q | disordered | |  |
| 297 | [rs1335162508](https://www.ncbi.nlm.nih.gov/snp/rs1335162508) | A459S/T | disordered | |  |
| 298 | [rs772418086](https://www.ncbi.nlm.nih.gov/snp/rs772418086) | R460S | disordered | |  |
| 299 | [rs1389840786](https://www.ncbi.nlm.nih.gov/snp/rs1389840786) | S461T | disordered | |  |
| 300 | [rs868512314](https://www.ncbi.nlm.nih.gov/snp/rs868512314) | T462N | disordered | |  |
| 301 | [rs776068920](https://www.ncbi.nlm.nih.gov/snp/rs776068920) | Q463H | disordered | |  |
| 302 | [rs1599271666](https://www.ncbi.nlm.nih.gov/snp/rs1599271666) | V466G | disordered | |  |
| 303 | [rs561258573](https://www.ncbi.nlm.nih.gov/snp/rs561258573) | T467I | disordered | |  |
| 304 | rs138380001 | R468S/C | disordered | |  |
|  | [rs200230807](https://www.ncbi.nlm.nih.gov/snp/rs200230807) | R468H/L | disordered | |  |
| 305 | [rs5498](https://www.ncbi.nlm.nih.gov/snp/rs5498) | K469E | disordered | |  |
|  | [rs766166027](https://www.ncbi.nlm.nih.gov/snp/rs766166027) | K469T | disordered | |  |
| 306 | [rs1242930269](https://www.ncbi.nlm.nih.gov/snp/rs1242930269) | V470M | disordered | |  |
| 307 | [rs1599271710](https://www.ncbi.nlm.nih.gov/snp/rs1599271710) | T471A | disordered | |  |
| 308 | [rs754970121](https://www.ncbi.nlm.nih.gov/snp/rs754970121) | V472M | disordered | |  |
| 309 | [rs1196540370](https://www.ncbi.nlm.nih.gov/snp/rs1196540370) | V474M | disordered | |  |
| 310 | [rs1453198780](https://www.ncbi.nlm.nih.gov/snp/rs1453198780) | L475F | disordered | |  |
| 311 | [rs369459423](https://www.ncbi.nlm.nih.gov/snp/rs369459423) | P477S/A | disordered | |  |
|  | [rs763594216](https://www.ncbi.nlm.nih.gov/snp/rs763594216) |  | |  |  |
| 312 | [rs5030400](https://www.ncbi.nlm.nih.gov/snp/rs5030400) | R474G/W | disordered | |  |
| 313 | [rs780437504](https://www.ncbi.nlm.nih.gov/snp/rs780437504) | R478Q | disordered | |  |
|  | rs5030400 | R478W |  | |  |
| 314 | [rs1480508060](https://www.ncbi.nlm.nih.gov/snp/rs1480508060) | T479H | disordered | |  |
| 315 | [rs781772466](https://www.ncbi.nlm.nih.gov/snp/rs781772466) | E480K | disordered | |  |
|  | [rs748177379](https://www.ncbi.nlm.nih.gov/snp/rs748177379) | E480V | disordered | |  |
| 316 | [rs777777071](https://www.ncbi.nlm.nih.gov/snp/rs777777071) | I481V | disordered | | transmembrane |
| 317 | [rs2145016489](https://www.ncbi.nlm.nih.gov/snp/rs2145016489) | V482I | disordered | | transmembrane |
|  | [rs749524458](https://www.ncbi.nlm.nih.gov/snp/rs749524458) | V482D/A | disordered | | transmembrane |
| 318 | [rs1371198155](https://www.ncbi.nlm.nih.gov/snp/rs1371198155) | I483V | disordered | | transmembrane |
| 319 | [rs868789554](https://www.ncbi.nlm.nih.gov/snp/rs868789554) | I484V | disordered | | transmembrane |
| 320 | [rs567569815](https://www.ncbi.nlm.nih.gov/snp/rs567569815) | V486A | disordered | | transmembrane |
| 321 | [rs187022571](https://www.ncbi.nlm.nih.gov/snp/rs187022571) | V487I | disordered | | transmembrane |
| 322 | [rs1472736379](https://www.ncbi.nlm.nih.gov/snp/rs1472736379) | A489T | disordered | | transmembrane |
| 323 | [rs775418785](https://www.ncbi.nlm.nih.gov/snp/rs775418785) | A490T/S | disordered | | transmembrane |
| 324 | [rs1382968890](https://www.ncbi.nlm.nih.gov/snp/rs1382968890) | M493V | disordered | | transmembrane |
| 325 | [rs763653983](https://www.ncbi.nlm.nih.gov/snp/rs763653983) | T495S | disordered | | transmembrane |
| 326 | [rs2040094725](https://www.ncbi.nlm.nih.gov/snp/rs2040094725) | A496T | disordered | | transmembrane |
| 327 | [rs2040094750](https://www.ncbi.nlm.nih.gov/snp/rs2040094750) | S499R | disordered | | transmembrane |
| 328 | [rs1292981351](https://www.ncbi.nlm.nih.gov/snp/rs1292981351) | T500S/A/P | disordered | | transmembrane |
|  | [rs372389375](https://www.ncbi.nlm.nih.gov/snp/rs372389375) | T500M | disordered | | transmembrane |
| 329 | [rs1599271948](https://www.ncbi.nlm.nih.gov/snp/rs1599271948) | Y501S | disordered | | transmembrane |
| 330 | [rs764821022](https://www.ncbi.nlm.nih.gov/snp/rs764821022) | Y503C | disordered | | transmembrane |
| 331 | [rs142682313](https://www.ncbi.nlm.nih.gov/snp/rs142682313) | R505C | disordered | |  |
|  | [rs755398718](https://www.ncbi.nlm.nih.gov/snp/rs755398718) | R505H/P | disordered | |  |
| 332 | [rs202205823](https://www.ncbi.nlm.nih.gov/snp/rs202205823) | R507W | disordered | |  |
|  | rs200510339 | R507L/Q | disordered | |  |
| 333 | [rs926467634](https://www.ncbi.nlm.nih.gov/snp/rs926467634) | I509T | disordered | |  |
| 334 | [rs771141539](https://www.ncbi.nlm.nih.gov/snp/rs771141539) | K510E | disordered | |  |
|  | [rs2145016631](https://www.ncbi.nlm.nih.gov/snp/rs2145016631) | K510R | disordered | |  |
| 335 | [rs1229166038](https://www.ncbi.nlm.nih.gov/snp/rs1229166038) | K511R | disordered | |  |
| 336 | [rs779108548](https://www.ncbi.nlm.nih.gov/snp/rs779108548) | Y512C | disordered | |  |
| 337 | [rs2040095223](https://www.ncbi.nlm.nih.gov/snp/rs2040095223) | R513K | disordered | |  |
| 338 | [rs374275620](https://www.ncbi.nlm.nih.gov/snp/rs374275620) | Q515L | disordered | |  |
| 339 | [rs2040095360](https://www.ncbi.nlm.nih.gov/snp/rs2040095360) | Q518K | disordered | |  |
| 340 | [rs2040095406](https://www.ncbi.nlm.nih.gov/snp/rs2040095406) | K519E | disordered | |  |
| 341 | [rs1432261188](https://www.ncbi.nlm.nih.gov/snp/rs1432261188) | G520R | disordered | |  |
|  | [rs1286797144](https://www.ncbi.nlm.nih.gov/snp/rs1286797144) | G520E | disordered | |  |
| 342 | [rs577755211](https://www.ncbi.nlm.nih.gov/snp/rs577755211) | T521P/A | disordered | |  |
|  | [rs1452847154](https://www.ncbi.nlm.nih.gov/snp/rs1452847154) | T521S | disordered | |  |
| 343 | [rs201409559](https://www.ncbi.nlm.nih.gov/snp/rs201409559) | P522S | disordered | |  |
|  | [rs1278808711](https://www.ncbi.nlm.nih.gov/snp/rs1278808711) | P522L | disordered | |  |
| 344 | [rs1358537165](https://www.ncbi.nlm.nih.gov/snp/rs1358537165) | M523K | disordered | |  |
|  | [rs776160824](https://www.ncbi.nlm.nih.gov/snp/rs776160824) | M523I | disordered | |  |
| 345 | [rs2040095671](https://www.ncbi.nlm.nih.gov/snp/rs2040095671) | P525T | disordered | |  |
|  | [rs761261553](https://www.ncbi.nlm.nih.gov/snp/rs761261553) | P525Q/L | disordered | |  |
| 346 | [rs1213697407](https://www.ncbi.nlm.nih.gov/snp/rs1213697407) | Q528K | disordered | |  |
| 347 | [rs376526495](https://www.ncbi.nlm.nih.gov/snp/rs376526495) | T530M | disordered | |  |
|  | [rs1225394164](https://www.ncbi.nlm.nih.gov/snp/rs1225394164) |  |  | |  |
