# Supplementary material for: Profiles of global mutations in the human intercellular adhesion molecule-1 (ICAM-1) shed light on population-specific malaria susceptibility
Source: BMC Genomics. 2023 Dec 13;24:773. doi: 10.1186/s12864-023-09846-9 (PMC10720214; doi:10.1186/s12864-023-09846-9)
Supplement: Supplementary file 2 — Supplementary Material 2 [file 12864_2023_9846_MOESM2_ESM.docx]

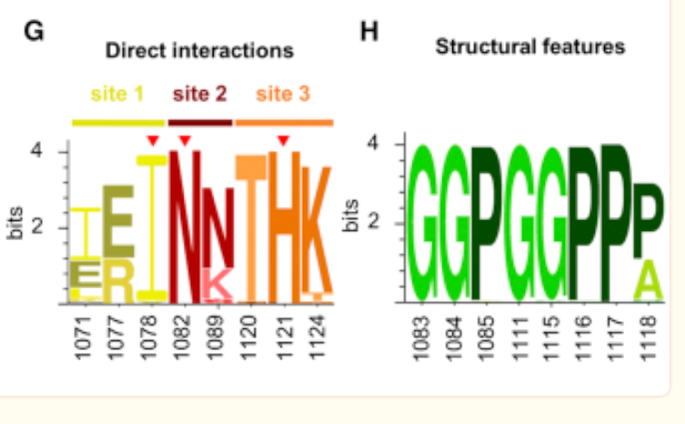

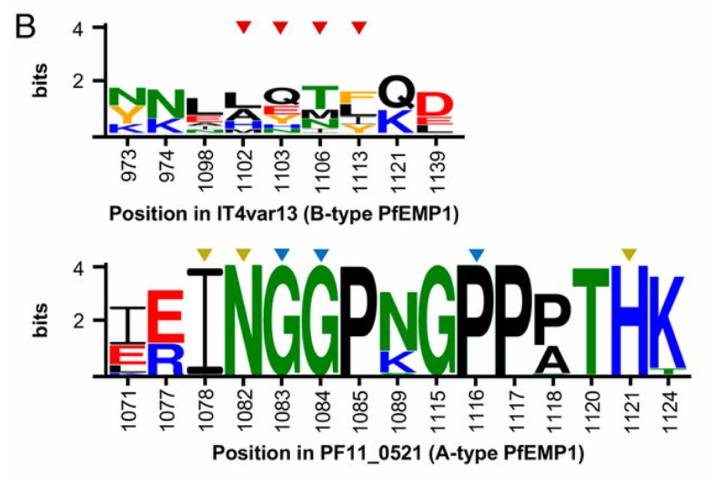


1. **B.**

**Supplementary file 2: A. Figure from Lennartz F, Adams Y, Bengtsson A, Olsen RW, Turner L, Ndam NT et al. Structure-Guided Identification of a Family of Dual Receptor-Binding PfEMP1 that Is Associated with Cerebral Malaria. Cell Host Microbe. 2017 Mar 8;21(3):403-414.** Sequence logo showing conservation of (G) residues that contact ICAM-1 and (H) residues important for the unusual architecture of the ICAM-1-binding site, based on 145 DBLβ domains predicted to bind ICAM-1. Red triangles, residues critical for direct interaction with ICAM-1. **B. Figure from Lennartz F, Smith C, Craig AG, Higgins MK. Structural insights into diverse modes of ICAM-1 binding by *Plasmodium falciparum*-infected erythrocytes. Proc Natl Acad Sci U S A. 2019 Oct 1;116(40):20124-20134**. Sequence logo showing all residues involved in ICAM-1 binding of A- or BC-type PfEMP1, based on 10 BC-type or 145 A-type DBLβ domains known or predicted to bind ICAM-1. Numbering is based on the IT4var13 and PF11_0521 sequences. Red triangles mark residues of IT4var13 that directly interact with ICAM-1. Yellow triangles mark residues of PF11_0521 that directly interact with ICAM-1. Blue triangles mark residues important for the conformation of the ICAM-1 binding site in PF11_0521.
